# Assessing tissue-specific gene expression of essential genes from human and mouse

**DOI:** 10.1093/bib/bbaf487

**Published:** 2025-09-24

**Authors:** Huiwen Zheng, Atefeh Taherian Fard, Jessica Cara Mar

**Affiliations:** Australian Institute for Bioengineering and Nanotechnology, Building 75, Corner of College Road and Cooper Road, University of Queensland, Brisbane, Queensland 4072, Australia; Australian Institute for Bioengineering and Nanotechnology, Building 75, Corner of College Road and Cooper Road, University of Queensland, Brisbane, Queensland 4072, Australia; Australian Institute for Bioengineering and Nanotechnology, Building 75, Corner of College Road and Cooper Road, University of Queensland, Brisbane, Queensland 4072, Australia

**Keywords:** essential gene, single-cell RNA-sequencing, multiomics, variability, tissue atlases

## Abstract

A gene is defined as essential when its functional loss compromises an organism's viability. Identifying essential genes is critical for identifying the components that regulate a biological system. Advances in gene editing techniques like CRISPR-Cas9 provide a capacity to interrogate a genome to elucidate the genes that are essential. However, these techniques are often applied for a single-cell line and rarely probed at a level of a tissue or organ. The recent availability of large-scale single-cell RNA-sequencing (scRNA-seq) atlases provides an unprecedented opportunity to investigate essential gene expression in a more comprehensive context. Our study leverages information from benchmarking datasets, single-cell tissue atlases, and databases of essential genes, to develop a method, scEssentials, that uses a statistical framework to investigate the robustness and specificity of essential genes across multiple cell types. Using scEssentials, mouse and human models showed consistently high expression and exhibited limited variability across more than 60 cell types. We demonstrate a substantial number of significantly correlated gene pairs that produce densely connected co-expression networks with functional annotation. Finally, we develop a score to quantify the relative essentiality of genes within scEssentials, further validating their significant association with gene mutation frequency and chromatin accessibility. Using ageing as an application, we demonstrate how scEssentials identifies robust gene expression profiles. Only one-fifth of scEssentials genes showed significant ageing-related differential expression among age groups. Collectively, the robustness of scEssentials serves as a reference for analysing scRNA-seq data and provides insight into the heterogeneous nature such as ageing.

## Introduction

Gene essentiality is critical for delineating the rules of a biological system because removing essential genes exposes the core genetic machinery that an organism requires to survive [1]. While essential genes are useful for understanding genetics, analysis of their gene expression profiles is valuable for functional genomics. This study develops a computational approach to explore the cell type-specific expression of essential genes for human and mouse. We leverage the diversity of publicly available information on essential genes and investigate this with single-cell tissue atlases to provide essential gene sets characterised by their expression level, gene expression variability, co-expression and other properties.

Typically, essential genes are identified without consideration of the specificity or context of cell or tissue types. The experimental methods used to identify essential genes are rarely conducted at single-cell resolution or tissue-wide manner. Advancements in molecular techniques have contributed to comprehensive databases of essential genes that have been validated for different organisms. Recently, the DEG15 database reported an increased number of eukaryotic and prokaryotic essential genes that were identified through transposon-insertion sequencing, CRISPR/Cas9, and single-gene knockout experiments [1]. Significant progress has been made in human essential genes, primarily due to studies that investigate the functions of essential genes in different donor- and patient-derived cell lines [2, 3]. Comparisons of human and mouse reveal minimal overlap in homologous essential genes, highlighting evolutionary changes in essential genes [4]. Additionally, discrepancies exist for identifying essential genes between *in vitro* cell lines and *in vivo* tissue samples [5]. Despite ongoing research and analysis, current knowledge of essential genes suggests it is conserved more at the functional level than at the individual gene level [4, 6].

To qualify as an essential gene, a gene must satisfy experimental validation of their essentiality e.g. through CRISPR /Cas9 or gene-knockout experiments. In systems biology, it is well known that some essential genes often overlap with housekeeping genes, ribosomes, and stably expressed genes (SEGs) due to their critical roles in survival. This overlap reflects the inherent redundancy in biological systems. For example, housekeeping genes are regarded as essential for cellular maintenance but not always stably expressed or essential across multiple species [7]. Examples exist where some ribosomes are essential for all organisms, but their expression patterns can be altered in tumour cells [8]. Unlike ribosomes and essential genes which are defined by functional properties, SEGs are mostly identified using statistical modelling of gene expression data. SEGs are often used for normalisation or standardisation, and some SEGs also play essential roles in cellular survival independent of the experimental conditions. The stability properties that lead to SEGs are not usually validated experimentally and hence a gene may function as a SEG only in the context of the dataset that was used to identify these genes [9]. Although essential genes may function as housekeeping genes with stable expression, it is important to distinguish between these definitions. For instance, studies in humans demonstrated that housekeeping genes exhibit greater evolutionary conservation than essential genes [10] whereas essential genes are linked to higher lethality and a weaker association with disease [11]. Therefore, our organism-specific essential gene lists may serve as a better reference to study the effects of gene expression changes in key biological processes.

With the identification of many essential genes established, recent efforts have focused on further exploring their functional properties in regulating biological systems. Li *et al.* summarised the topological characteristics of essential genes in protein–protein interaction networks and related biological information [12]. Previous studies have investigated the characteristics of essential genes at the transcriptome level and revealed their important functional roles in the epigenetic landscape of normal cells [4]. These studies provide a foundation for characterising essential genes at different omics levels. Understanding how essential genes are expressed within a single-cell type is valuable because cell type identity is driven by gene expression programs. Since only a subset of genes is expressed in any given cell type, examining the consistency of essential gene expression across diverse cellular contexts provides critical insights into their roles within the organism. Such analyses could further inform computational approaches to identify and prioritise essential genes critical for cellular survival, offering a refined framework for understanding their functional importance in maintaining organismal viability. Advances in sequencing technologies have enabled transcriptome analysis at single-cell resolution. However, the low capture efficiency and sequencing limitations of scRNA-seq often result in missing genes within the data. The presence of these missing genes may stem from technical constraints of scRNA-seq technologies and platform-specific effects should also be considered in the analysis. Additionally, *in vivo* assays may introduce higher noise compared to *in vitro* data, making experimental context critical to interpreting essential genes. Therefore, investigating the characteristics of essential genes at the cell type level with largescale single-cell atlases like Tabula Muris (TM) and Tabula Sapiens (TS) is crucial [13, 14]. To the best of our knowledge, this study is the first evaluation of essential genes at the single-cell level in human and mouse. Our study aims to provide gene sets that satisfy the computational properties of essential genes across cell types in normal conditions. These gene sets represent utility for future perturbation studies where the loss of gene expression for these specific genes is more likely to lead to severe consequences.

In this study, we first measure which genes are likely to be essential based on properties like robustness, corresponding biological functions, and co-expression connectivity. We use over 100 cell types and 10 different sequencing platforms to identify a set of essential genes that satisfy quality control and reliability detection for single-cell RNA-seq data using a computational approach termed scEssentials. These preranked genes can be applied to any scRNA-seq dataset to either normalise the data or identify gene expression changes. Next, we evaluated the characteristics of the scEssentials genes and their robustness in multiomics data. High expression levels and robust essentiality scores were highly associated with other molecular levels. Lastly, we demonstrated the stable expression of the scEssentials genes during ageing to show how scEssentials can contribute knowledge to this dynamic and heterogeneous universal biological process.

## Methods

### Developing a computational method, scEssentials, to refine essential gene lists

Original lists of essential genes identified for human and mouse were downloaded from the DEG15 database [1], including 2354 mouse essential genes and 8988 human essential genes from different experimental techniques. Two benchmarking datasets were used to evaluate the reliability of expression by assessing technical variation as scRNA-seq data typically have higher dropout rates (See Supplementary Note for details, Supplementary Table 1). These essential genes were further assessed by >50 cell types from TM and TS to confirm detectability and stability *in vivo*. Like the previous step, we removed essential genes with low detectability and stability. In total, 733 and 1969 essential genes for mouse and human, respectively, were denoted as scEssentials genes.

### Data normalisation, marker detection, and stably expressed gene signatures

*SCTransform* [15] was applied to normalise the data by the mice/donor effect for TS and TM. Differential expression analysis was performed with *MAST* [16] in a 1-vs-all fashion within each tissue to identify cell type-specific markers. The highly variable gene signatures were identified by *vst* in *Seurat (v4.0)* [17] for each cell type.

The human SEGs (precomputed) and housekeeping genes were derived from three studies identified through statistical modelling and meta-analysis from large datasets [9, 18, 19]. Specifically, only cytosolic ribosomal genes were selected from *Deeke et al* as these genes showed high expression and strong stability in bulk RNA-seq data (Supplementary Table 2). The hypergeometric test of the overlaps between scEssentials genes and other gene lists with respect to random gene lists can be found in the Supplementary Note.

### Constructing an essentiality score

An *in silico* essentiality score was defined for each scEssentials gene from two characteristics, noncell type-specificity and robustly high expression. We leveraged *PanglaoDB* (27_Mar_2020) [20] to quantify if any of the scEssentials genes were cell type-specific markers (Supplementary Figs 1–5). A scEssentials gene with no evidence as a cell type-specific marker and with high detectability across cell types was up-weighted where a higher ES implied greater importance towards that organism (See Supplementary Note).

### Cell type similarity based on essential genes and visualisation

Pearson correlation was applied to measure the relationships among sequencing methods and cell types from TM and TS based on the scEssentials genes’ expression. The correlation plots that illustrated the similarities of different aspects of essential genes were constructed by corrplot *(v0.9)* [21].

### Measuring gene expression variability in scRNA-seq data

We used *scran* (*v1.20.1*) to measure the cell-to-cell variability with mouse/donor ID as a covariate in the regression model as it shows the highly accurate calculation of cell-to-cell variability in scRNA-seq data [22, 23]. Since the estimates of biological variability from *scran* are based on the residual values from generalised linear regression models, genes with negative values represent those that have biological variability that are lower than the expected values.

### Gene pairs correlation level and co-expression networks based on scEssentials at the cell-type level

A modified Spearman correlation statistic assessed co-expression between gene pairs in scRNA-seq data using *correlatePairs* function from *scran* [22]. As excessive zeroes affect the correlation, genes with non zero expression values for >90% of each cell type were excluded. *P*-values were corrected for multiple testing using Benjamini-Hochberg method and statistical significance was set at 0.05. The difference in the number of gene pairs was assessed using the proportion test. The number of significantly correlated gene pairs varied substantially between different random gene lists, so only the top 100 most correlated gene pairs (both positively and inversely correlated) were extracted from each cell type (or the maximum number if the total number is >10 and <100, otherwise the cell type was removed). The Kruskal-Wallis test was applied to determine the level of significant gene pairs’ correlation coefficients to compare scEssentials and random gene lists for each cell type. To compute the correlated scEssentials gene pairs, boxplots for the absolute R values and histogram representing the mean absolute correlation coefficients for each cell type were combined by *ggpubr (v0.4.0)* package [24].

Co-expression networks were constructed for scEssentials genes and a random sample set based on significantly correlated gene pairs (*P* < .01). Betweenness and normalised node degree of each network were calculated by *igraph (v1.2.6)* [25]. Subsequently, a set of robustly correlated gene pairs was aggregated if they were significantly correlated in more than half of the total cell types and the network was plotted by *igraph*. A fast greedy modularity optimisation algorithm was applied to the shared network to identify communities, and related functions were computed against the Gene Ontology (GO) database.

### Gene sets from curated pathway databases

To measure the proportions of essential genes in the curated pathways, we used the Hallmark, KEGG, Reactome, and WikiPathways gene lists obtained from the *msigdbr (v 7.4.1.)* package [26]. The number of essential genes found in each pathway was compared to the random gene list. The Kruskal-Wallis *test* was used to assess whether the number of essential genes in the pathway was more in the scEssentials genes by comparing this against 10 random gene lists.

### Gene damage index (GDI)

The *in silico* GDI score is an estimate of a gene’s mutation damage that has accumulated in the general population under a comprehensive multilevel analysis [27]. A high GDI score corresponds to frequently mutated genes that are more likely to cause inherited and rare diseases but unlikely to have a significant and lethal consequence. The Kruskal-Wallis test was applied to test whether the Phred-scaled GDI indexes were similar between scEssentials genes and random gene lists.

Additionally, comparisons between the scEssentials expression variability differences in three GDI categories corresponding to low (91 genes), medium (1728 genes), and high GDI (39 genes) were conducted using the Kruskal-Wallis test. To mitigate the impact of unequal sample sizes, downsampling was performed on the number of genes in the medium GDI category to 39 through the downsampling method in caret (v6.0–90) [28]. This procedure was repeated 10 times.

### Chromatin accessibility level inferred from single-cell assay for transposase-accessible chromatin sequencing (scATAC-seq)

To investigate the chromatin accessibility of the scEssentials genes, we used cell types in marrow tissue from TM and mouse scATAC-seq atlases [29]. We included five cell types with >100 cells including hematopoietic progenitors, B cells, macrophages, monocytes, and immature B cells to be consistent with the cell-type selection in the TM. Only the promoter transcription start sites that overlapped the processed peaks were included. Spearman correlation was applied to measure the relationship between the chromatin accessibility level and the essentiality score for genes within each cell type.

### Transcription factors (TF) identification

*AnimalTFDB 3.0* was used to search for mouse TFs in the scEssentials [30]. To calculate the enrichment of TFs that were overlapping in the scEssentials gene set, we applied the hypergeometric test with all protein-coding genes as the background gene set. In total, 24,351 mouse protein-coding genes were extracted from the Mouse Genome Informatics database [31]. Significance was calculated by *Phyper* in R.

### Modelling the scEssentials gene expression changes in ageing

Only mice from 3-month, 18-month, and 24-month that have been sequenced by FACS-smartseq2 methods from Tabula-Muris-Senis (TMS) [32] were included in the analysis to reduce data sparsity. We excluded cell types that had <100 cells. To understand how essential genes change during ageing, we fitted a gene-specific generalised linear regression model by analysing the interaction between age and cell type while controlling sex as a covariate. For each gene $i$, the model is


$$ {y}_i\sim{sex}_i+{age}_i\ast{celltype}_i $$

where ${sex}_i$ (male and female) was corrected in the model and interaction term ${age}_i\ast{celltype}_i$ measured the effect of age groups (3-month, 18-month, and 24-month) depending on different cell types for each gene $i$. P-values were corrected for multiple testing using the Benjamini-Hochberg method. Genes that showed significant changes across age groups, were then further investigated for expression changes under the effect of different cell types. Genes that showed no significant difference in age groups, were then further investigated for any significant interactions of their gene expression values with different cell types. Barplots and boxplots were used to assess performance of scEssentials genes under the age covariate and their effect on cell types.

### Pathway over-representation analysis

The pathway over-representation analysis was performed on the scEssentials genes that significantly changed during ageing via *clusterProfiler (v4.0.5)* using GO, with genes from TM and TS as the universe [33]. The top 10 significantly enriched pathways based on P-values were retained.

### Statistical analysis and visualisation of data

Random gene lists were sampled from the full data matrix with the same number of essential genes and sampling was repeated 10 times. Plots were generated through *ggplot2 (v3.5.1)* unless stated with the seed 123,441. The BH adjustment method was applied to correct for multiple testing and statistical significance threshold was 0.05.

## Results

### Developing a computational method, scEssentials, that identifies essential genes from single-cell atlas data

**Figure 1:**
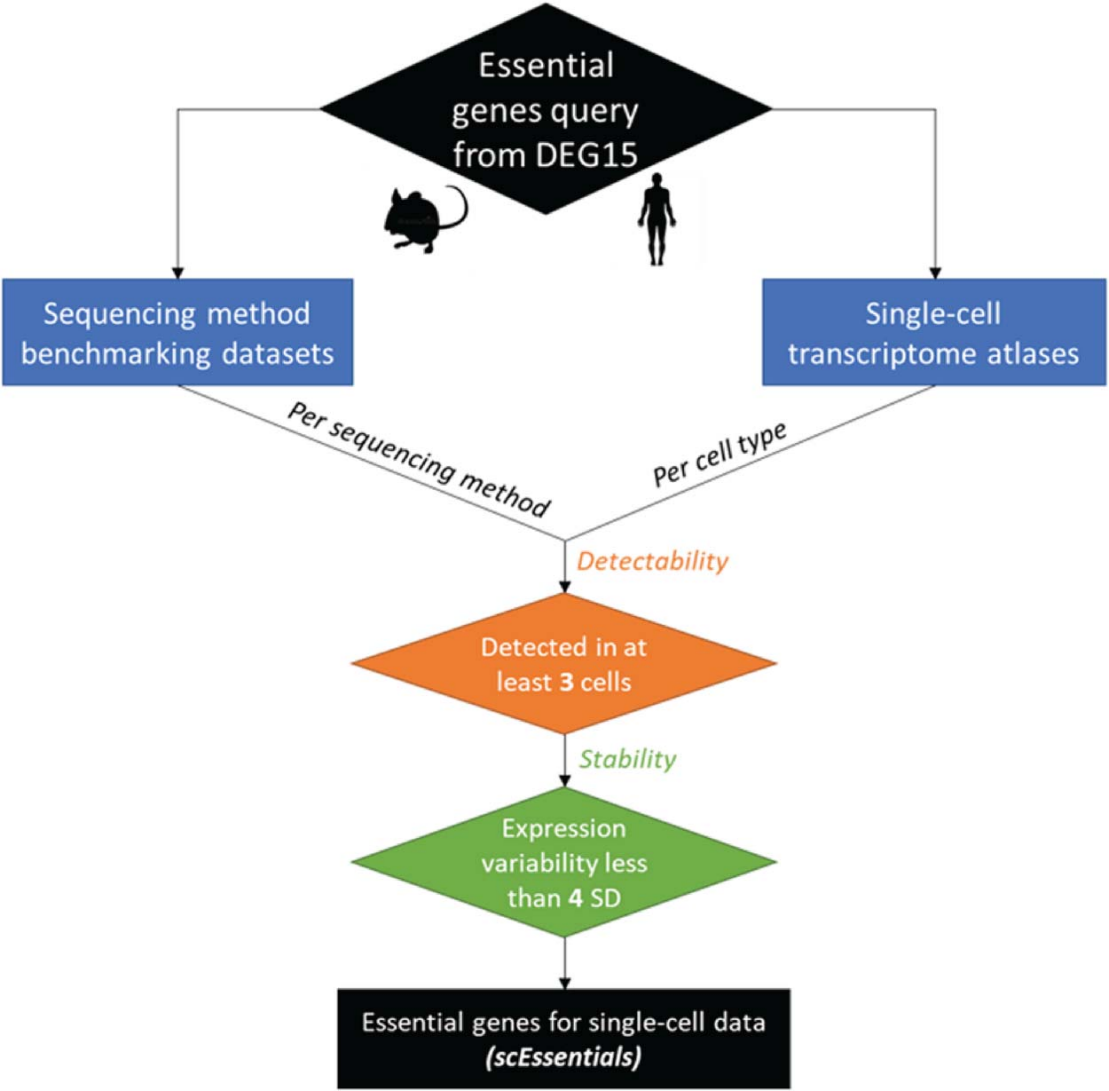
Overall workflow to determine essential genes for single-cell data (scEssentials). Experimental-derived essential genes were queried from the DEG 15 database [1] for mouse and human, respectively. To ensure the detectability of scEssentials genes, we select the essential genes which have at least three nonzero reads in every sequencing method that was sequenced only with cell line samples and also at least three cells in each cell type in TM/TS and retain the intersection of genes for downstream analysis.

We first considered the detectability and stability of essential genes to ensure that any results from scEssentials were reliable (Fig. 1). On average 80% of essential genes were detected across different sequencing methods for mouse and human datasets. For non-UMI methods, a higher rate of >90% of essential genes were detected (Supplementary Figs 1–5). Conversely, decreased detectability was found in TM (70%) across 68 cell types and substantially less in TS (64%) across 53 cell types, which may have been due to greater heterogeneity in sequencing variation for human compared to mouse samples. Additionally, highly variable essential genes were removed either due to a lack of technical (sequencing methods) or biological (cell types) detectability. Interestingly, we observed higher variation in essential genes from technical rather than biological sources (Supplementary Fig. 6). Finally, we retained 733 and 1969 essential genes for mouse and human, respectively and these genes are henceforth noted as scEssentials genes. The correlation plots of scEssentials gene expression demonstrated high correlations among cell types in both TM ($mean\ r$ = 0.75) and TS ($mean\ r$ = 0.80) (Supplementary Fig. 7), where each tissue-cell-type was closely clustered with the same cell types rather than tissue type.

### scEssentials genes are highly expressed in all cell types with minimal overlap with cell type-specific markers

Essential genes are known to have higher expression than other genes [4]. We confirmed this feature in scEssentials genes at the cell type level as these genes showed significantly higher expression among all cell types in TM and TS data compared to random gene lists (Supplementary Fig. 8). This supports the observation that scEssentials genes had higher expression values based on tissue-level RNA-seq data [4]. Additionally, scEssentials are expressed in a higher percentage of cells among all cell types (Supplementary Fig. 9).

**Figure 2:**
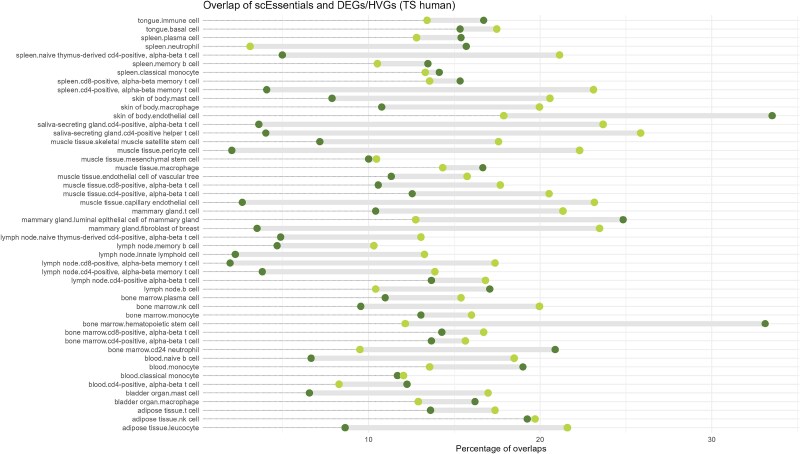
Investigating the overlap between scEssentials genes and two types of gene sets, cell type specific DEGs and HVGs in TS (human). Dark green represented the overlap with DEGs and light green represented the overlap with HVGs.

A key consideration in analysing scRNA-seq data is the identification of cell type-specific markers that include differentially expressed genes (DEGs) and highly variable genes (HVGs). We used a hurdle model to determine DEGs and examined the overlap with scEssentials genes in any cell type [17]. As expected, most of the cell type-specific DEGs shared minimum overlap with the scEssentials genes for mouse and human datasets (median 5% (33/733 scEssentials) in TM; median 11% (217/1969 *scEssentials*) in TS). We compared scEssentials genes with two cell type marker databases *PanglaoDB* [20] and *CellMarker 2.0* [34] to further measure cell type specificity (Supplementary Fig. 3). Although we found 119 mouse and 175 human scEssentials genes were identified as cell-type markers, only about one-sixth of cell types in the *PanglaoDB* were represented with the cell types available in TS. Similarly, the HVGs and scEssentials genes with TM data showed an even smaller overlap (median 4%). However, the HVGs shared a higher proportion with scEssentials genes with TS data (median 27%) (Fig. 2, Supplementary Fig. 10-11), which might result from variability associated with the TS donors. Overall, the higher number overlap of scEssentials genes with DEGs and HVGs in TS likely reflects the increased heterogeneity of human compared to mouse data.

In contrast to HVGs, ribosomal genes, SEGs, and housekeeping genes were also compared to scEssentials genes because of their related characteristics. In general, most of the SEGs and housekeeping genes were curated by different statistical algorithms or meta-analyses of related literature which often shows inconsistency and method dependency. Comparisons of three gene lists with scEssentials genes showed an overlap of around 56% of the total gene lists regardless of the size of the different gene lists (Supplementary Fig. 12A). Interestingly, the majority of ribosomal genes (82%) overlapped with at least one gene list, as they had high expression levels and critical roles in cell growth and proliferation [35]. In addition, scEssentials genes showed a significant overlap with housekeeping genes, ribosomal genes, and SEGs, with genes in this overlap involved in RNA catabolic process and splicing functions (Supplementary Fig. 12B).

Finally, we proposed an *in silico* essentiality score (ES) for scEssentials genes that incorporates both gene detectability and marker gene sensitivity. Particularly, the top-ranked genes were mostly ribosomes and involved in apoptosis for human and mouse datasets, which agreed with the previous study that summarised the essential genes' potential categories [4]. In summary, although human and mouse scEssentials genes shared only approximately half of their homologous genes, they were highly consistency in enriched indispensable biological pathways. Notably, mouse scEssentials genes had more shared transcription factors compared to human which had more ribosomal genes (Supplementary Fig. 13).

### scEssentials gene pairs are highly and consistently correlated in various cell types

**Figure 3:**
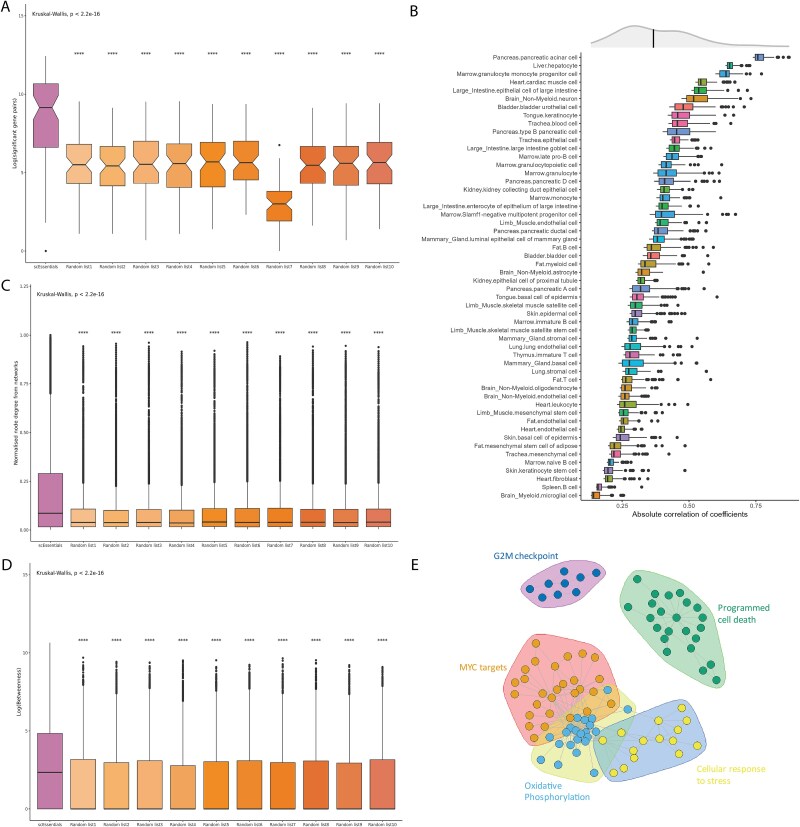
Analysing the correlation of mouse scEssentials gene pairs against 10 random gene lists. Boxplots illustrated (A) the number of significant gene pairs; (B) the top 100 most correlated scEssentials gene pairs across cell types (coloured by tissue origin); (C) network betweenness; (D) network normalised node degrees constructed from scEssentials and random gene lists. (*P*-value ^*^ < .05, ^**^ < .01, ^***^, < .001, ^****^ .0001). (E) A core network from significantly consistently correlated scEssentials gene pairs with four communities.

We next investigated co-expression of scEssentials genes to identify functional relationships from this gene set. To avoid spurious correlations due to high sparsity, we used a modified Spearman correlation analysis with a permutation test to determine co-expression between gene pairs [22]. We observed a substantial increase in the number of correlated gene pairs with scEssentials genes compared to random genes for mouse (Fig. 3A) and human (Supplementary Fig. 14A).

Interestingly, even though the sizes were different, the number of significant co-expressed gene pairs was proportional between the scEssentials genes and randomly sampled gene lists. This indicates that the levels of tight co-regulation were distinct across different cell types regardless of the genes used for this measurement. For each cell type, the scEssentials genes obtained a consistently higher number of significantly correlated gene pairs for 84% of the cell types (57/68). Interestingly, cell types that did not show a higher number of correlated gene pairs in scEssentials mostly consisted of precursor cells from different tissue origins.

Furthermore, we assessed the degree of correlation in scEssentials genes compared to randomly sampled genes. The top 100 most correlated gene pairs from each gene list were extracted and compared with the coefficients. Consistently, we observed correlation coefficients in the scEssentials genes that were significantly higher in the value of the correlation coefficients compared to those obtained for 10 random gene lists in all cell types, except for two progenitor cells in marrow tissue (Supplementary Fig. 15, 66 out of a total of 68 cell types). Furthermore, we investigated the degree of scEssentials gene pairs’ correlation across cell types. The absolute coefficients for the selected correlated scEssentials gene pairs had mean absolute coefficient value at 0.36 (Fig. 3B). Notably, such correlation values were significantly higher than the ribosomal genes mean absolute correlation values (0.24), mostly across all 68 cell types (Supplementary Fig. 16). Unlike the consistent expression pattern for individual scEssentials levels, the correlation range between scEssentials varied among cell types. Interestingly, the most tightly correlated cell types were specialised epithelial cells, particularly, pancreatic acinar cells, hepatocytes, and cardiac muscle cells. Overall, scEssentials correlation coefficients showed consistently high values and higher cell-type variations than at the single gene level.

Finally, we constructed the co-expression network from the common significantly co-expressed scEssentials gene pairs across cell types to assess the functional relationship of these genes. Betweenness and normalised node degree were used to measure network connectivity. Eventually, on average, networks constructed by scEssentials genes across cell types showed significantly higher node degrees and the nodes (genes) in each network showed higher betweenness as compared to random gene lists, which indicated that the scEssentials correlation network tended to have closer connections possibly to maintain functional roles (Fig. 3C and D, Supplementary Fig. 14B and C). Furthermore, we identified a core co-expression network from common co-expressed gene pairs in >30 cell types, including 86 nodes (genes) and 227 vertices (correlations). Five community structures were identified by a fast greedy modularity optimisation algorithm, and each community was functionally related to apoptosis, oxidative phosphorylation, and cell maintenance (Fig. 3E).

### scEssentials genes displayed a low frequency of somatic mutation i.e. associated with a low level of gene expression variability

Given the stability that scEssentials genes possess, it is expected that such stability is present at other molecular levels like stably encoded proteins [4]. To investigate scEssentials genes at other molecular levels, we used the gene damage index (GDI), which quantifies the mutation frequency of protein-coding genes in the general population [27]. scEssentials genes showed a significant correlation with low gene damage prediction and decreased normalised GDI value compared to other protein-coding genes (Supplementary Table 2). Lower GDI indicated that the less frequently mutated genes, which are more likely to be disease-causing, represent critical roles of the scEssentials genes at the genetic level to maintain regular cellular activity.

**Figure 4:**
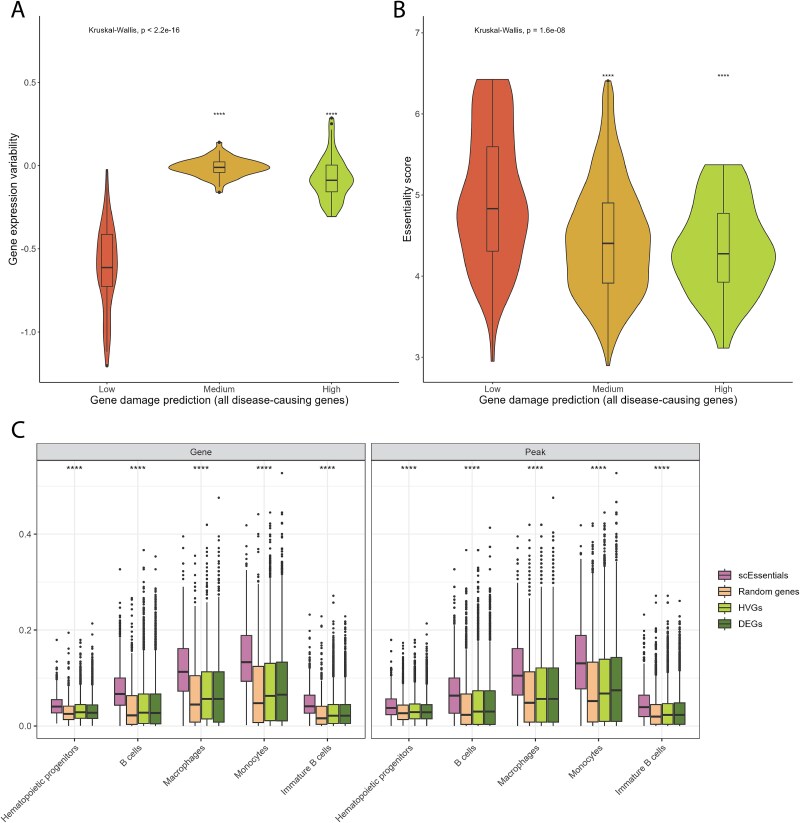
Analyses of mouse scEssentials genes with gene mutation index and chromatin accessibility level. (A) Violin plot showed gene expression variability within low, medium, and high-risk groups. (B) Boxplot showed the ES score difference among groups. (C) Chromatin accessibility level for five cell types from scATAC-seq atlas in scEssentials genes and a random gene list, measured by peaks and gene level (*P*-value ^*^ < .05; ^**^ < .01; ^***^ < .001, ^****^ < .0001).

Next, we interrogated the link between mutation frequency and gene expression variability. GDI values were categorised into low, medium and high-risk levels based on thresholds from the original paper [27]. Interestingly, significantly low gene expression variability was associated with low GDI on average, which aligns with the interpretation that genes with increased expression stability are associated with lower risk of mutation frequency in disease-causing genes. Although there was no significant mean difference in gene expression for the medium and high GDI groups, the high GDI group demonstrated significantly increased cell-to-cell variability (Fig. 4A). Specifically, we observed a commonly increased level of gene expression variability in genes with medium damage and high damage as compared to low damage for over 42% of cell types (22/53) (Supplementary Fig. 17A). Additionally, genes in the low GDI group showed an increased number of more stable genes (negative values) than more variable genes (positive values) as compared to other groups. The increased gene expression variability illustrated the potential impact of somatic mutation burden on stochastic heterogeneous gene expression. We found that most of the SEGs were due to a set of ribosomal genes whereas the top variable genes were associated with regulation of cell death. However, when comparing the scEssentials genes with high GDI to non-scEssentials genes in the high GDI group, the former group showed significantly reduced gene expression variability in >77% of cell types (41/53) (Supplementary Fig. 17B). This result emphasises that the stability of the scEssentials gene expression level was robust even though they possess high risks for damage from mutations in disease.

Lastly, we analysed the relationship between ES and the normalised GDI score to assess the ability of the ES value to capture mutation frequency. Low GDI scEssentials found significantly high ES as compared to medium and high GDI groups (Fig. 4). Furthermore, ES values were significantly inversely correlated with the normalised GDI score across all cell types, with a median correlation coefficient − 0.4 (*P < .05;*  Supplementary Fig. 17C), suggesting that the mutation frequency can be well-captured by ES.

### scEssentials genes showed a significantly higher level of chromatin accessibility and a higher number of transcription factors

Other than gene mutations, gene expression is regulated by epigenetic factors such as chromatin packaging and accessibility. To investigate the scEssentials genes' active regulation, we selected five cell types from the scATAC-seq atlas [29] that shared the greatest number of cell types with TM data (Supplementary Fig. 18A). Compared to the random gene list and other reference gene lists, the peaks that intersected with the transcription starting site of scEssentials genes showed significantly higher levels of accessibility (Fig. 4C). Furthermore, we aggregated multiple peaks annotated to one gene to compare at the gene level. scEssentials genes showed further increased degrees of chromatin accessibility level compared to random gene lists at peak level (Fig. 4C).

To evaluate whether the ES value can capture the level of chromatin accessibility, we computed the correlation between ES and chromatin accessibility for each cell type. As expected, ES significantly correlated with accessibility (Supplementary Fig. 18B). Furthermore, scEssentials genes had a significantly higher number of transcription factors (TFs) responsible for controlling gene expression (Supplementary Fig. 18C). Taken together, we demonstrated that scEssentials genes possessed coordinated regulation at the epigenetic level to maintain stable gene expression and the accuracy of ES values at different molecular levels such as genetics and epigenetics.

### scEssentials genes showed high involvement in the curated pathway databases

**Figure 5:**
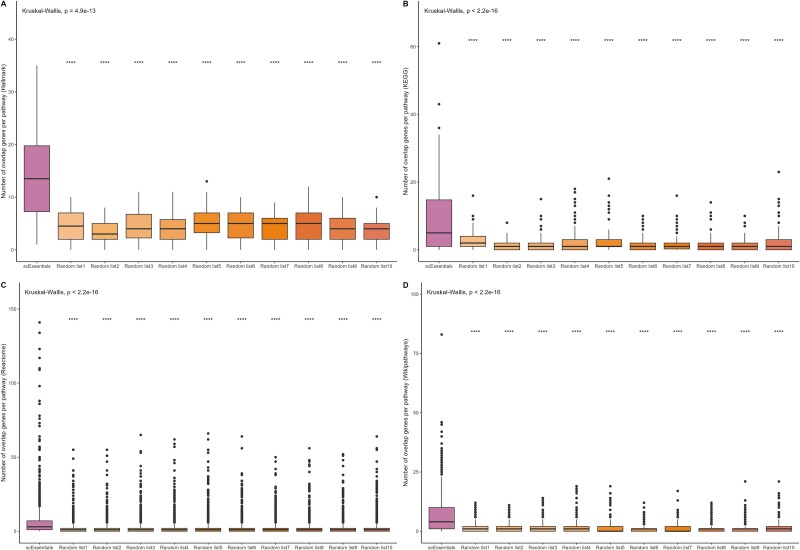
Percentage overlap with two pathway databases (A) Hallmark, (B) KEGG, (C) Reactome, and (D) WikiPathways. scEssentials genes and 10 randomly sampled gene lists were plotted in the boxplot (*P*-value ^*^ < .05; ^**^ < .01; ^***^ < .001, ^****^ < .0001).

Biological pathways are comprised of interactions between genes that produce a product in a cell. Therefore, genes in a pathway are more likely to modify cellular functions than genes not in that pathway. By leveraging the comprehensive pathway knowledge of KEGG, Hallmark, Reactome, and WikiPathways, we investigated the involvement of scEssentials genes in more than 20 k biological pathways [36–39]. We assessed the overlap between the scEssentials genes and each pathway to demonstrate the importance of scEssentials genes relative to random genes. For each pathway, we observed a significantly increased contribution of scEssentials genes compared to randomly sampled gene lists, indicating the fundamental roles that scEssentials genes play in maintaining biological functions (Fig. 5). Notably, essential genes were mostly identified by experimental functional screening or knockdown experiments with well-characterised genes [40], which may create an over-annotation effect in the curated databases. However, the application of 10 randomly sampled gene lists addresses this effect and supports the evidence that scEssentials genes function as a core component among various biological pathways. To further explore how scEssentials genes contribute to biological pathways differently from other reference gene sets such as housekeeping genes, we examined their overlap with genes involved in apoptotic pathways from the KEGG database. Notably, we identified unique scEssentials genes such as *Trp53* within the apoptotic pathway, which is a key regulator of cellular stress responses i.e. indispensable for cell survival [41].

### Dysregulation of scEssentials genes showed cell-type-specificity in ageing

Since transcriptional dysregulation is commonly observed in ageing in a tissue-specific and cell-type-specific pattern [42–45], we next investigated whether scEssentials genes change during ageing. We applied a gene-specific generalised linear regression model to >130 cell types to investigate the interaction between cell types and age for scEssentials genes from Tabula Muris Senis (TMS) database [32]. Surprisingly, we found that only 25% (184/733) of scEssentials genes’ expression was dysregulated with age, indicating the importance of scEssentials genes regardless of the ageing phenotypes. Additionally, these genes were found to be significantly associated with DNA repair and apoptotic pathways (Supplementary Fig. 19A). These pathways are the signaling cascades triggered by DNA damage, which is the primary cause of ageing [46]. We further compared the ES score between the significantly dysregulated scEssentials genes and nonsignificant ones. Interestingly, we did not observe a significant difference in the ES score for the genes that were associated with age (Supplementary Fig. 19B).

**Figure 6:**
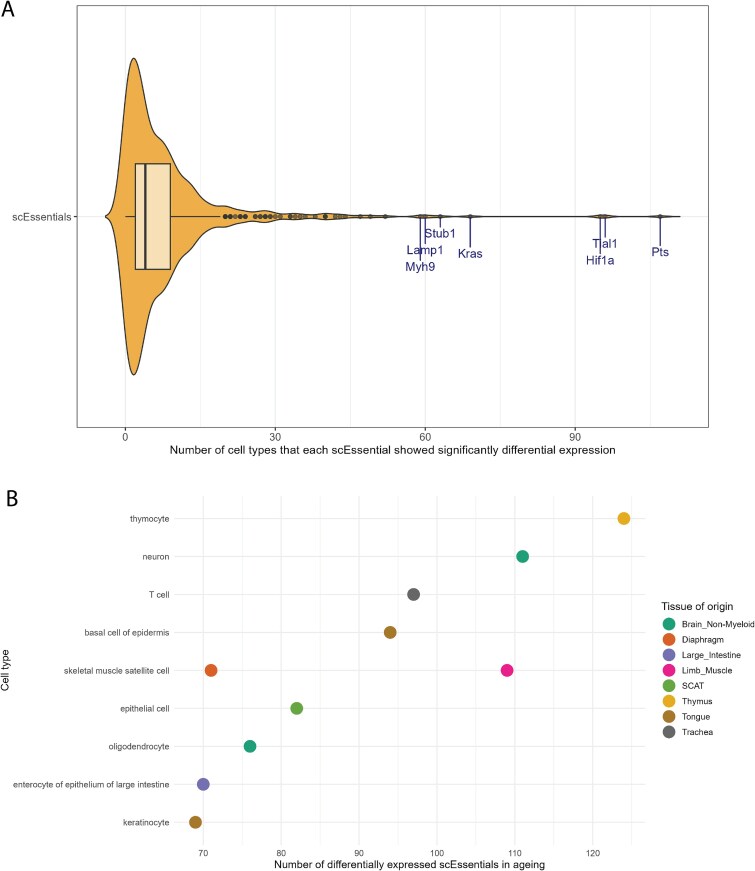
Dysregulation of scEssentials genes among cell types in ageing. (A) Violin plot for each scEssentials gene that was differentially expressed. Genes with the top number of significant changes among cell types are highlighted. (B) Dot plot illustrating the top 10 cell types with the highest number of scEssentials genes that showed significant change in ageing. Each dot was coloured by tissue of origin.

Additionally, we explored the modification of scEssentials genes at the cell type level for the remaining scEssentials genes (nonsignificantly changed with age covariate). Inspecting gene expression changes across age groups for each cell type, we observed that scEssentials genes had dysregulation that only occurred in a small proportion of cell types (median 4/136) while *Pts*, *Tial1, Hif1a, Kras, Stub1,* and *Lamp1* were the most actively changing scEssentials genes, especially in the 24-month group (Fig. 6A). Interestingly, we found that the top cell type-specific genes whose expression profiles had a significant interaction term between age and cell type (*Pts*, *Kras*, and *Stub1*) were also classified as housekeeping genes, but not as SEGs, further highlighting their biological relevance. To understand whether ageing-related changes are more associated with certain cell types, we investigated the frequency of scEssentials genes that showed significant dysregulation in ageing within each cell type. Strikingly, all cell types were found to have at least one scEssentials gene that significantly changed with age (Supplementary Fig. 19C), with median of scEssentials genes per cell type at 22 across the three age groups. Fig. 6B shows the top 10 cell types with the highest number of significant scEssentials genes associated with age. Skeletal muscle satellite cells, T cells, and epithelial cells were identified regardless of different tissue origins, which have shown profound functional deterioration in ageing [47, 48]. Additionally, high numbers of differentially expressed scEssentials genes were found that closely interacted with brain cell types like neurons and oligodendrocytes. Studies have suggested that changes in oligodendroglia impact their neurofunctional roles and are highly associated with declining brain function in ageing [49].

## Discussion

Our work extends existing knowledge of essential genes to single-cell resolution at different molecular levels for mouse and human. Leveraging benchmarking datasets with more than 10 sequencing platforms and large-scale mouse and human transcription atlases, we identified robust scEssentials gene signatures across 60 cell types that can be used as a reference for single-cell transcriptomic studies. These genes are involved in basic cellular functions regardless of cell type. We confirmed this with a comprehensive study on scEssentials genes at multiple levels of resolution, starting with single gene, gene pairs, and multigene as well as multimolecular levels, to improve the understanding of gene essentiality in heterogeneous cellular functions.

We showed that scEssentials genes have high expression, high proportions of expression, and low cell type-specificity in both human and mouse, which can serve as an independent negative control for evaluation studies. However, a distinguishing feature of scEssentials genes that separates them from housekeeping genes and/or SEGs is that a level of gene expression variability is observed in scEssentials genes. Although such variation is significantly lower than in nonessential and nonhousekeeping genes, it may reflect the transcriptional fluctuations that occur under normal perturbations that an organism experiences to maintain key functions specifically in human samples.

Our study unlocks the cell state specificity of scEssentials genes by investigating gene-pair correlation. Genes work together to co-regulate activity; thus, gene–gene correlations capture the underlying dynamics of gene regulation. Overall, a significantly higher number of correlated gene pairs and higher correlation in scEssentials genes compared to nonessential genes were observed and further support the critical co-regulation that occurs beyond a single gene level. Moreover, the consistently high co-expression patterns in cell types in specialised epithelial cells suggest that the high regulatory constraints of scEssentials genes pertain to differentiated cells. Our analysis further expands the knowledge that such tightly co-regulated and essential processes in specialised epithelial cells can be identified with different tissue origins, indicating scEssentials gene pairs are critical regulators to identify cell type specificity.

The consensus co-expression network constructed from various cell types has shown critical roles in pathways related to cell energy, cell cycle, and cell maintenance. Incorporating consensus co-expression patterns into scRNA-seq clustering methods has already been developed to improve the accuracy of identifying enriched pathways [50, 51]. While these methods use different statistical approaches to find robust and denoised co-expression patterns across batches or biological conditions, it may be beneficial to include inherently stable co-expressed gene signatures from scEssentials genes to improve clustering of scRNA-seq data.

The essentiality score we developed showed strong associations with the degree of gene mutation and chromatin accessibility, demonstrating the robustness of this score at multimolecular levels. The criticality that it illustrates allows for generating a flexible and robust quantification for any analytical application in a data-dependent manner. Moreover, it provides evidence for the robust transcription process for the scEssentials genes with tight chromatin regulation.

Additionally, our analysis demonstrates the greater involvement of scEssentials genes in multiple pathways that are curated by two different pathway databases. Together with previous findings that essential genes are more likely to be located as hub genes in protein–protein interaction networks [4], our result demonstrates crucial roles in pathway network location. It illustrates that scEssentials genes are the common regulatory genes to support complex biological processes, such as DNA repair and apoptosis. Notably, numerous studies have identified DNA repair defects accelerate ageing by triggering stem cell exhaustions, resulting in increased apoptosis and ultimately impairing their normal proliferation and differentiation ability [52]. Thus, this reinforces the characteristics of scEssentials in maintaining cell viability. Furthermore, scEssentials genes can be used as one of the key sources when mitigating the highly overlapping genes among different pathways, which has been shown to improve pathway overrepresentation analysis [53].

To further explore the robust gene expression of scEssentials genes, we investigated the scEssentials gene expression changes in the heterogeneous ageing process. We found that over 80% of the scEssentials genes do not significantly change their expression among the three age groups. This analysis demonstrates the robustness of scEssentials gene expression in the accumulated stress conditions in ageing. In contrast, the consistently differentially expressed scEssentials genes among different cell types have already been linked to dysregulation of the ageing human skeletal muscle (e.g. *Pts*) or triggering upregulation of other ageing factors like p53 (e.g. *Tial1*) [54, 55]. Therefore, such dysregulated scEssentials genes may be more likely to cause phenotypic changes in the cell types. Our analysis found that muscle satellite cells had most of the differential expression of essential genes in ageing. This result supports previous findings that muscle stem cell dysfunction is a major contributor to the decline in the regenerative potential of muscle tissue during ageing [56]. In line with the stem cell decline phenomenon which is known as a hallmark of ageing, we showed that stem cell exhaustion is a universal consequence in multiple stem-like cell types and occurs in multitissue and multiorganismal ageing [48].

Overall, our study demonstrates the significant role of scEssentials genes in maintaining cellular functions in multiple layers. Leveraging atlas-size scRNA-seq databases, we show robust, consistent, and high gene expression of scEssentials that can be used in different applications to analyse complex processes supported by the essentiality score. In addition, scEssentials genes offer a potential biological reference for normalising spatial transcriptomics data, ensuring accurate interpretation beyond technical variability. Given the fundamental roles that scEssentials genes play, we demonstrate how subtle changes in scEssentials genes in ageing may cause significant consequences, especially in stem cells of different tissue origins.

**Key Points**

- This study refined sets of essential genes specific to humans and mice, which are robust and applicable in various scRNA-sequencing data across sequencing technologies and cell types.
- The essential genes exhibit remarkable stability and high expression across single gene, gene pair, and pathway levels, significantly outperforming that observed in 10 simulated gene sets.
- Further investigation of essential gene characteristics at the multi omics level reveals a significantly lower gene mutational burden and a greater extent of accessible chromatin regions.
- Our novel scEssentials score effectively captures gene essentiality as evidenced by high correlations with gene expression variability and the gene damage index, an external metric that quantifies mutation frequency.
- The essential genes remain stable during the ageing process with significant expression changes primarily observed in stem cells origin from different tissues.

## Supplementary Material

Supplementary_Figures_and_Tables_Revised_Clean_26Aug2025_bbaf487

SupplementaryNote_Revised_Clean_26Aug2025_bbaf487

## Data Availability

TM and TS scRNAseq data with raw count matrix and cell type annotation is available in Figshare: https://figshare.com/articles/dataset/Tabula_Muris_Senis_Data_Objects/12654728 and https://figshare.com/articles/dataset/Tabula_Sapiens_v2/27921984. The multi platform sequencing data of mESCs is available at GEO accession code GSE75790. The multi platform sequencing data of human donors is available at GEO accession code GSE118767. The multi platform sequencing data of mixed-species human, mouse and dog reference sample data is available at GEO accession code GSE133549. Mouse scATAC-seq data can be downloaded from https://atlas.gs.washington.edu/mouse-atac/data with cell type annotation. Human and mouse scEssentials gene and the code to generate the plots and processing scheme can be found at the GitHub repository https://github.com/huiwenzh/scEssentials.
