# Supplementary material for: Assessing tissue-specific gene expression of essential genes from human and mouse
Source: Brief Bioinform. 2025 Sep 24;26(5):bbaf487. doi: 10.1093/bib/bbaf487 (PMC12459261; doi:10.1093/bib/bbaf487)
Supplement: Supplementary_Figures_and_Tables_Revised_Clean_26Aug2025_bbaf487 [file supplementary_figures_and_tables_revised_clean_26aug2025_bbaf487.docx]

**Supplementary Figures and Tables**

Supplementary Table 1. Details of the benchmarking datasets used to measure the scEssentials gene expression detectability across sequencing methodology.

| **Organism** | **Number of cells** | **Sequencing method** | **Cell type** | **3' or full-length** | **Reference** |
| --- | --- | --- | --- | --- | --- |
| **Mouse** | 71 | CELseq2 | Embryonic stem cells | 3' | (1) |
|  | 76 | Dropseq |  | 3' |  |
|  | 65 | MARSeq |  | 3' |  |
|  | 84 | SCRBseq |  | 3' |  |
|  | 130 | Smartseq |  | full-length |  |
|  | 157 | Smartseq2 |  | full-length |  |
| **Human** | 1439 | 10X Chromium (10X_LLU) | B lymphocytes | 3' | (2) |
|  | 3296 | 10X Chromium (10X_NCI) |  | 3' |  |
|  | 3273 | 10X Chromium (10X_NCI_M) |  | 3' |  |
|  | 241 | Fluidigm C1 HT ( C1_FDA_HT) |  | 3' |  |
|  | 66 | Fluidigm C1 ( C1_LLU) |  | full-length |  |
|  | 596 | Takara Bio ICELL8 (ICELL8_PE) |  | full-length |  |
|  | 600 | Takara Bio ICELL8 (ICELL8_SE) |  | full-length |  |

Supplementary Table 2. The number of scEssentials and other protein-coding genes that had been classified based on gene mutation index. The *Chi-square test* was applied to determine the relationship (p <0.05).

|  | **scEssentials** | **Non-scEssentials** |
| --- | --- | --- |
| **Low** | 111 | 316 |
| **Medium** | 4203 | 14121 |
| **High** | 112 | 695 |


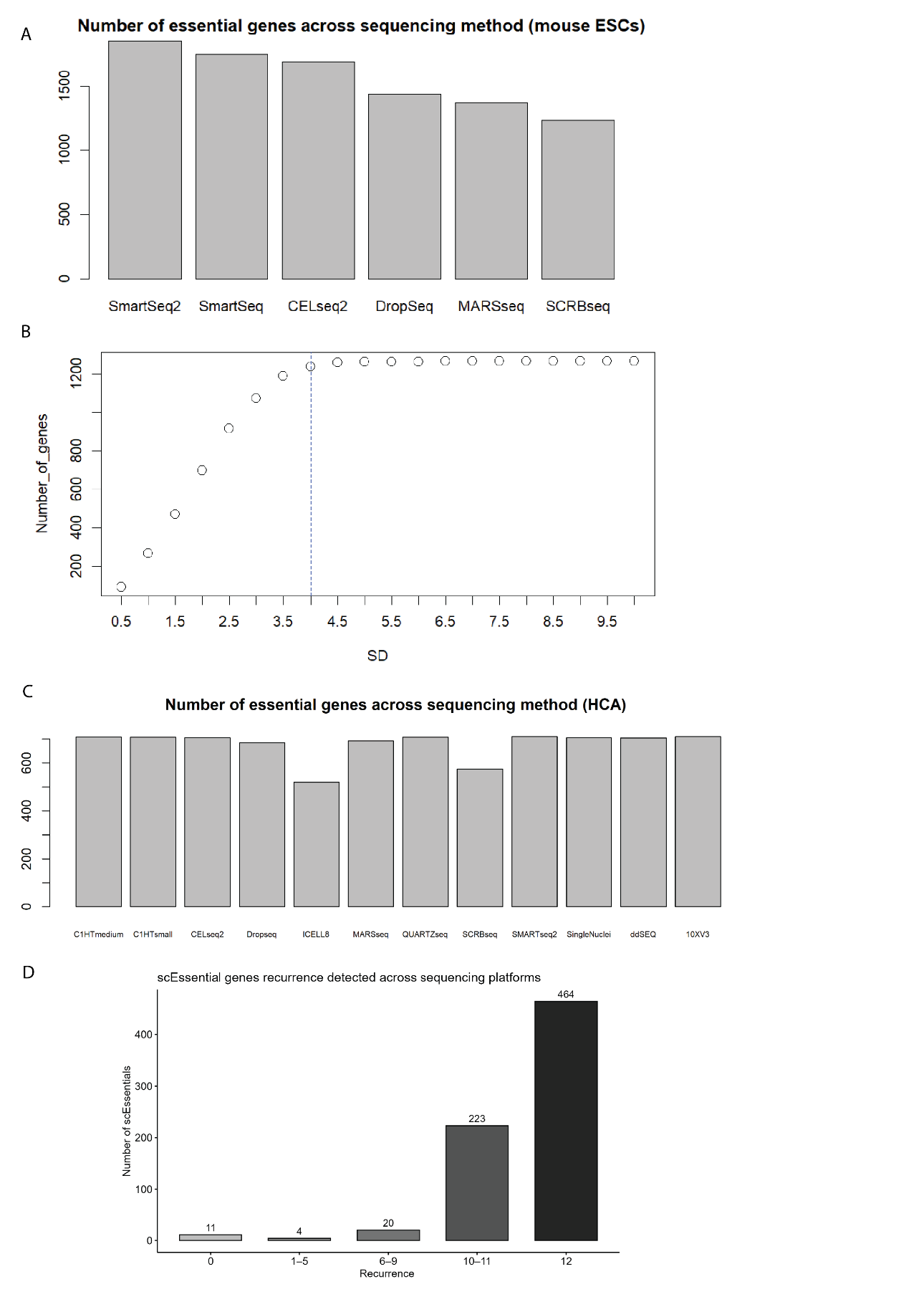


Supplementary Figure 1. Detectability and variations of mouse essential genes across different sequencing platforms. A) The number of mouse essential genes that had expression across six sequencing methods in mESCs (1). B) Sensitivity analysis to investigate an appropriate threshold based on the standard deviation (SD) of essential genes’ expression across sequencing for protocols. The blue dashed lines represented 4 times the overall SD and demonstrates where an elbow is achieved, hence, 4 was adopted as the threshold for filtering genes based on instability across protocols. C) The number of mouse essential genes in the multi-species sample across sequencing protocols (3). D) Number of scEssential genes identified across different single-cell protocols. Each bar represents the count of scEssentials consistently detected in a given number of protocols.


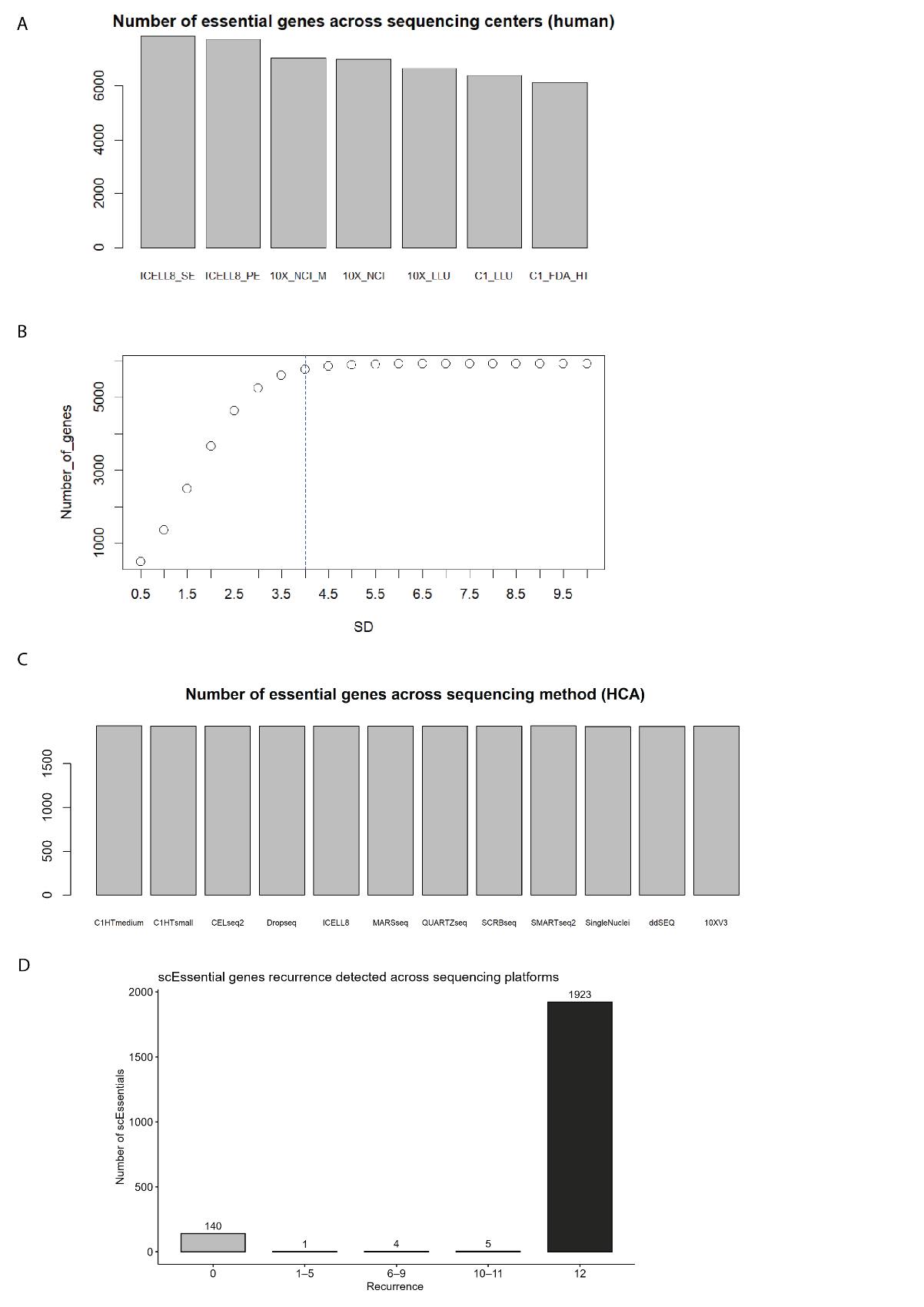


Supplementary Figure 2. Detectability and variations of human essential genes across different sequencing platforms. A) The number of human essential genes that had expression across seven sequencing methods in B cells. B) The elbow plot demonstrated the standard deviation (SD) of essential genes’ expression across sequencing protocols, with the number of genes removed. The blue dashed lines represented 4 times the overall SD. C) The number of human essential genes in the multi-species sample across sequencing protocols (3). D) Number of scEssential genes identified across different single-cell protocols. Each bar represents the count of scEssentials consistently detected in a given number of protocols.


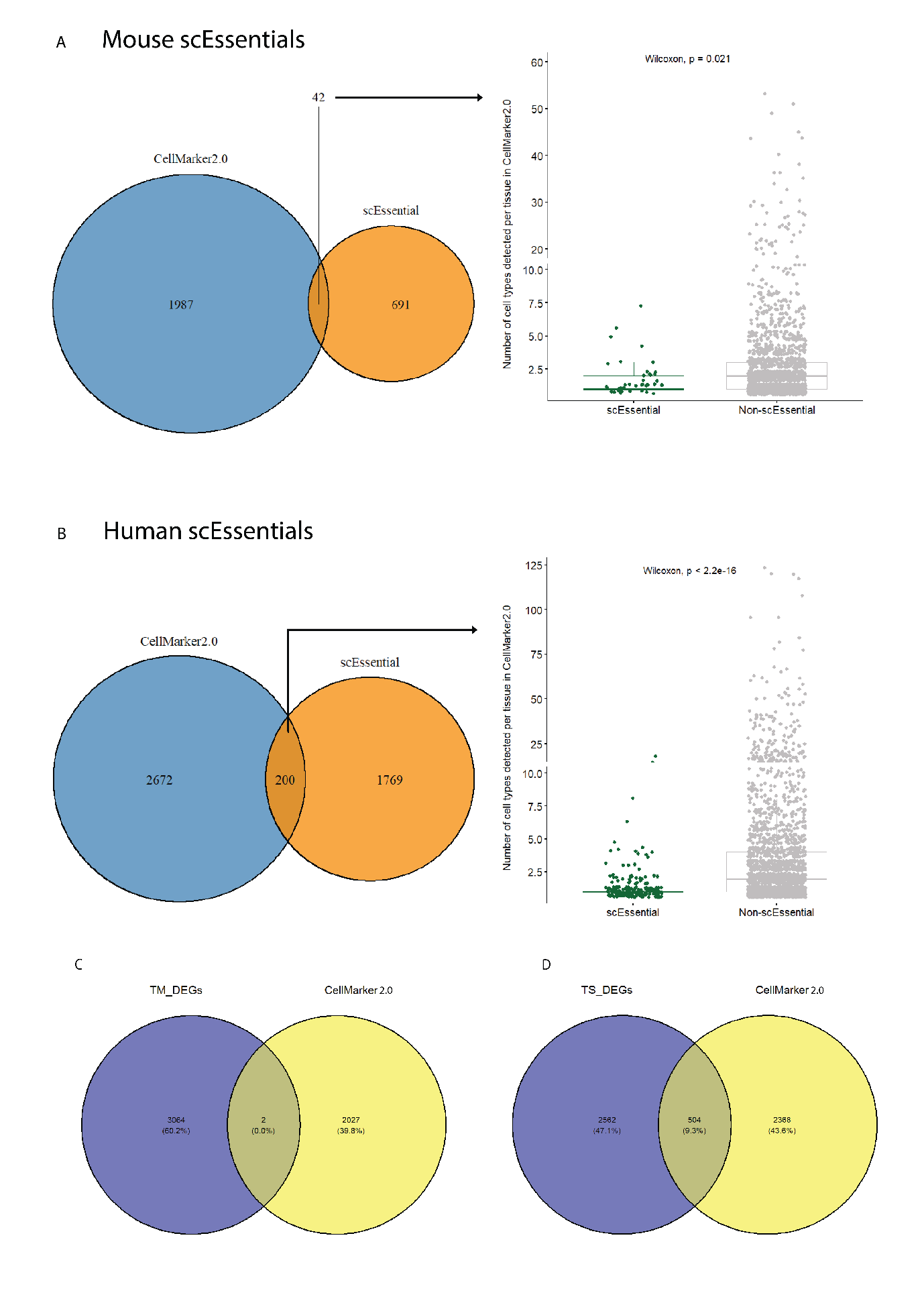


Supplementary Figure 3. Comparison of scEssentials genes and cell type-specific markers from CellMarker 2.0. **A)** Venn diagram showis the overlap between mouse scEssentials genes and cell type-specific markers. The overlapping scEssentials genes exhibit significantly fewer occurrences in the CellMarker 2.0 database compared to non-scEssentials (Wilcoxon rank-sum test). **B)** Venn diagram showing the overlap between human scEssentials and cell type-specific markers. Similar to the mouse data, the overlapping human scEssentials have significantly fewer occurrences in the CellMarker 2.0 database compared to non-scEssentials (Wilcoxon rank-sum test). **C)** Venn diagram showed the overlaps between CellMarker 2.0 and DEGs from TM data. **D)** Venn diagram showed the overlaps between CellMarker 2.0 and DEGs from TS data.


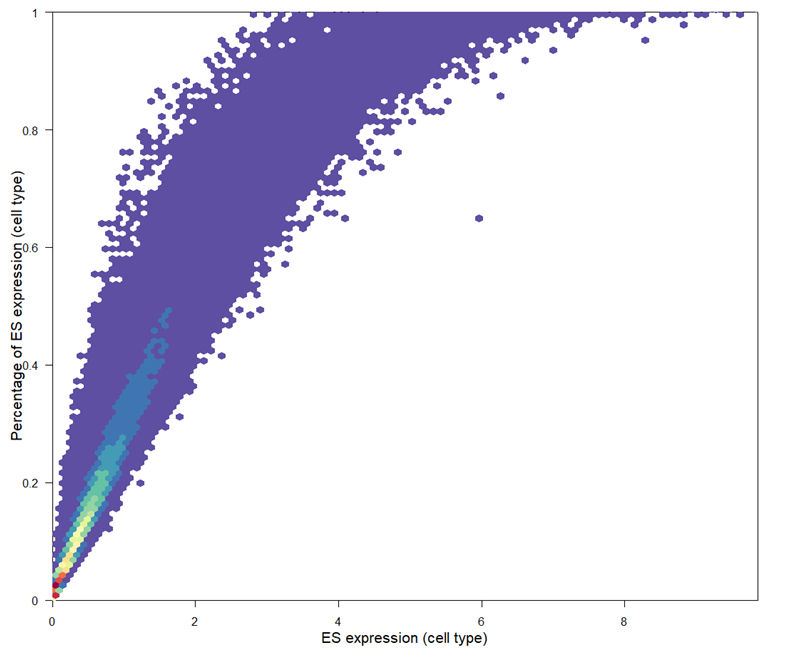


Supplementary Figure 4. Correlation of average expression and percentage of the cell’s expressed for scEssential genes. The high correlation illustrated the percentage of cell’s expression captured the high level of expression characteristics while providing a wider comparison range.


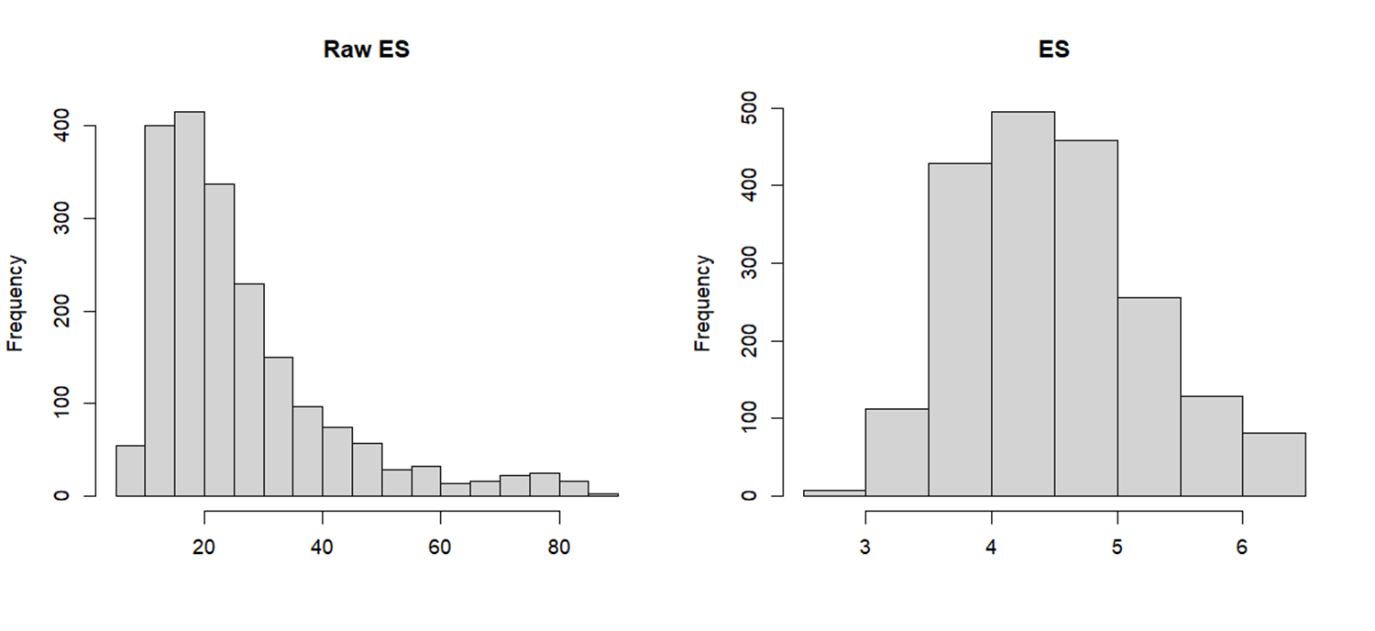


Supplementary Figure 5. The distribution of raw ES and ES values for scEssentials. On the left, raw ES distribution showed right-skewness and with substantial value difference. To mitigate such skewness, the logarithm2 transformation was applied to raw ES score.


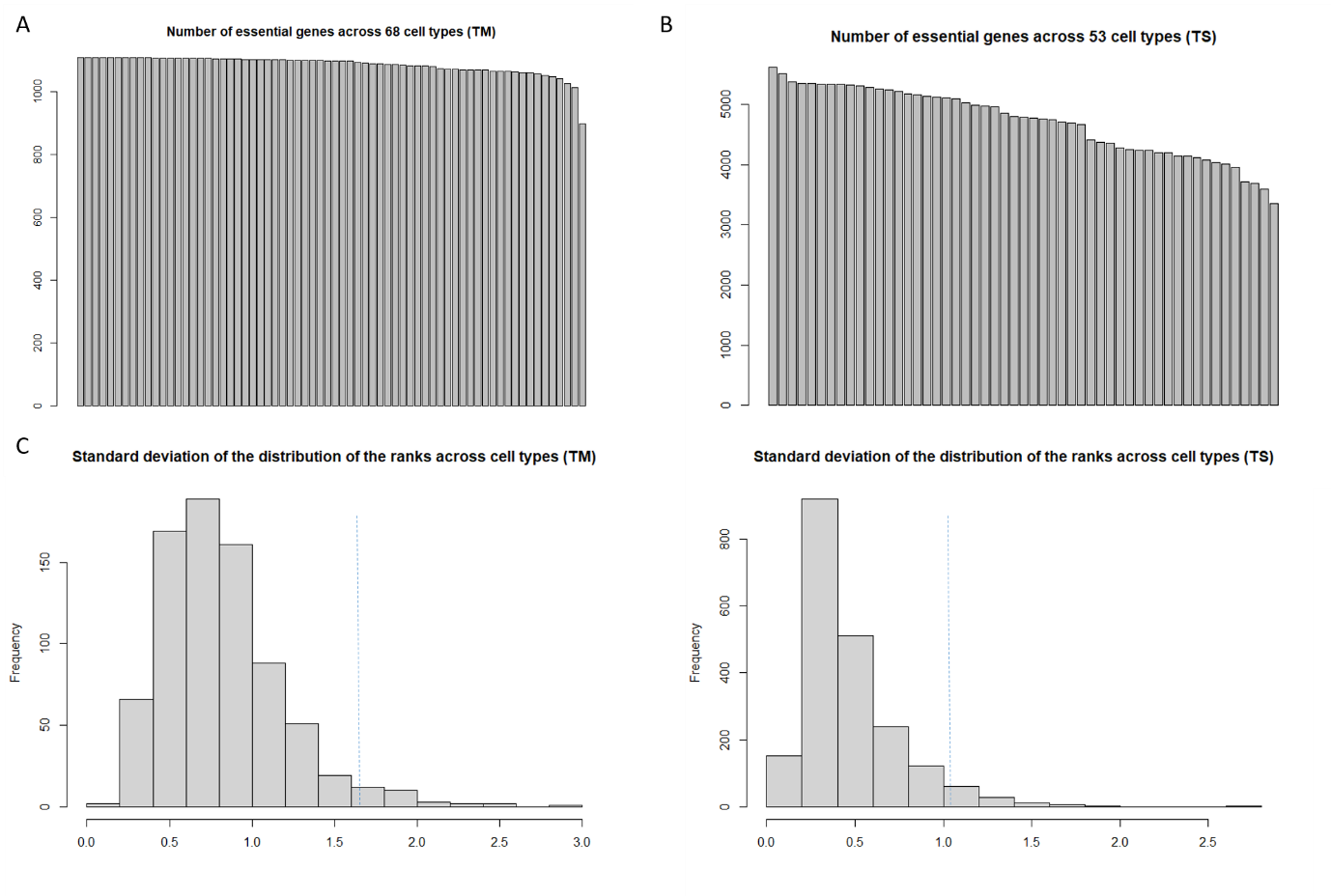


Supplementary Figure 6. Detectability and variations of essential genes in more than 60 unique cell types. The number of essential genes that had expression across A) 68 cell types in Tabula Muris (TM) and B) 53 cell types in Tabula Sapiens (TS) (4). Figure C and D demonstrated the standard deviation (SD) of each essential gene’s expression across cell types in TM and TS. The blue dash lines represented 4 times the overall SD.


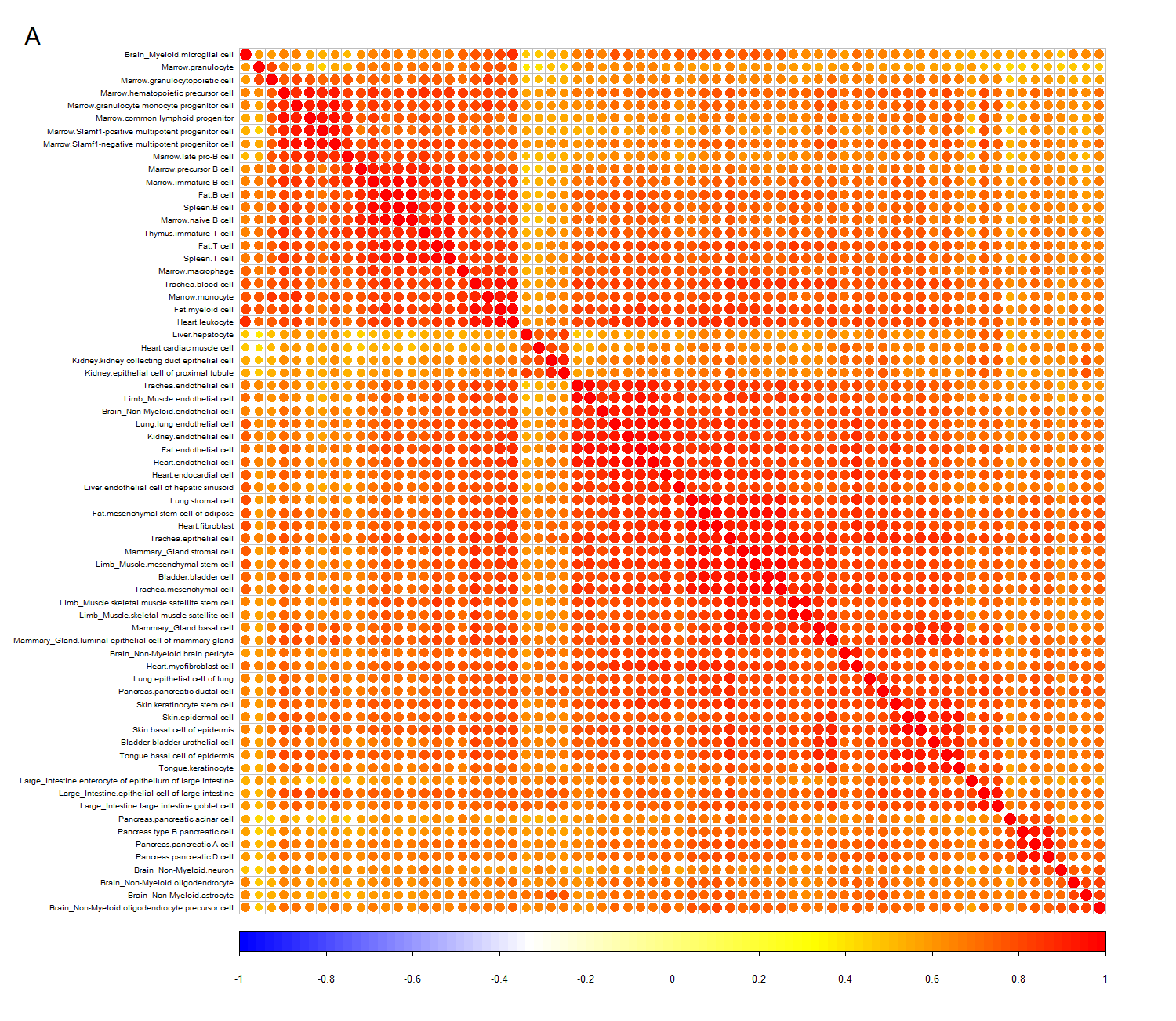


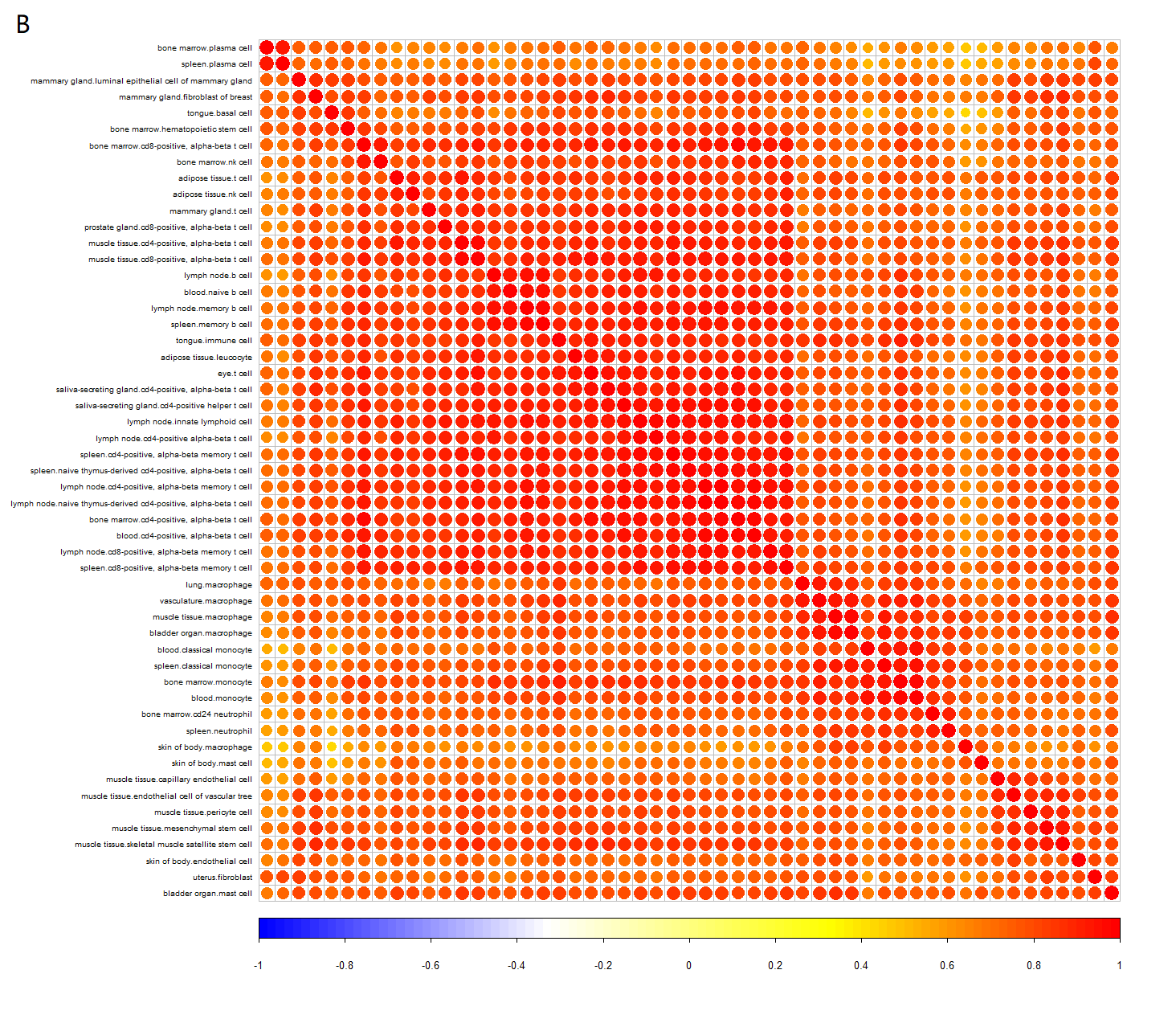
Supplementary Figure 7. Correlation heatmap across cell types based on scEssential genes. Pearson correlation was applied to measure the similarities among cell types. By performing the correlation with expression values, the heatmaps showed non-cell-type-specific patterns with an average higher correlation A) with the TM dataset; B) with the TS dataset. But the correlation reduced with clear cell-type-specificity when the scEssentials expression ranking replaced the expression value.


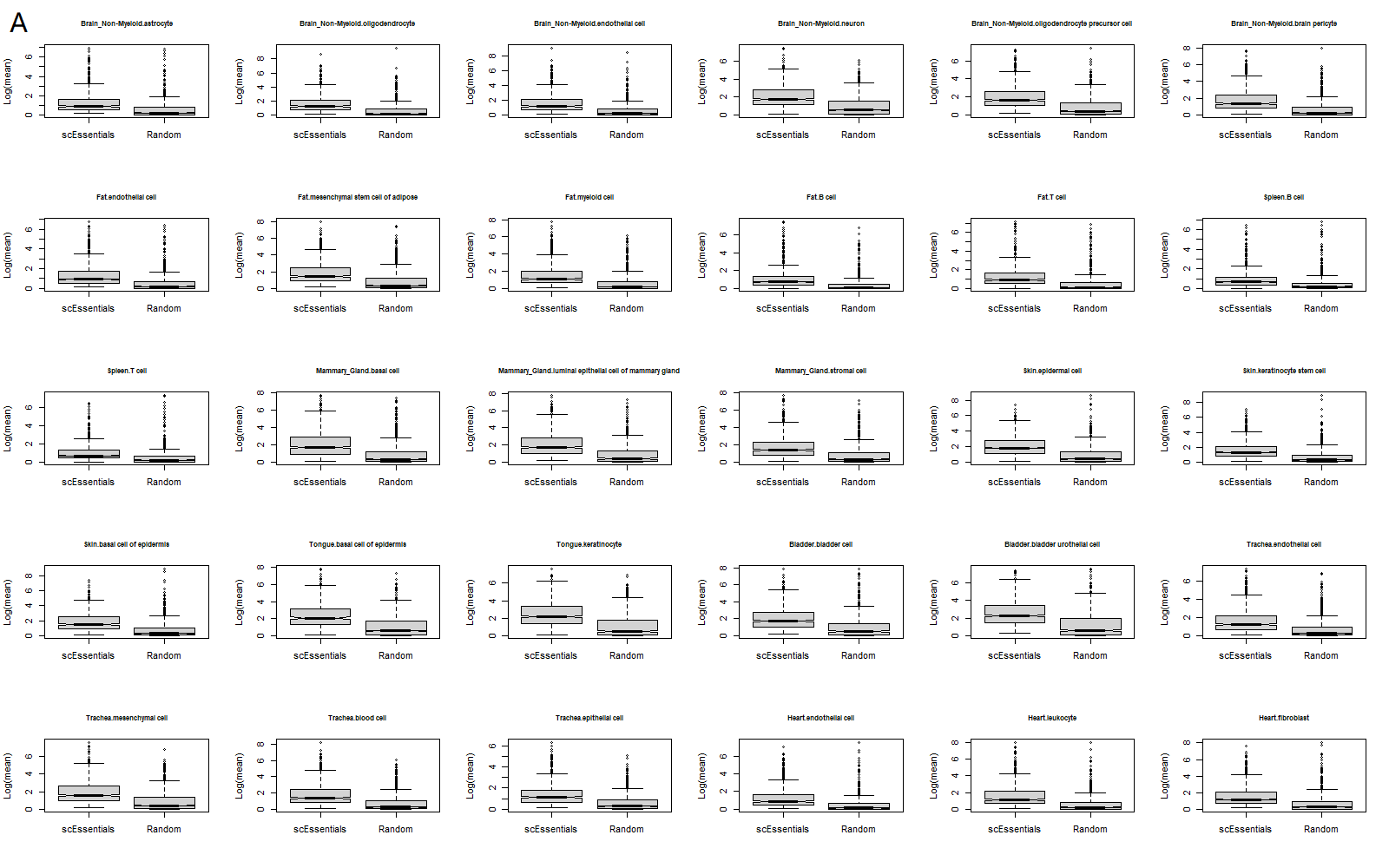


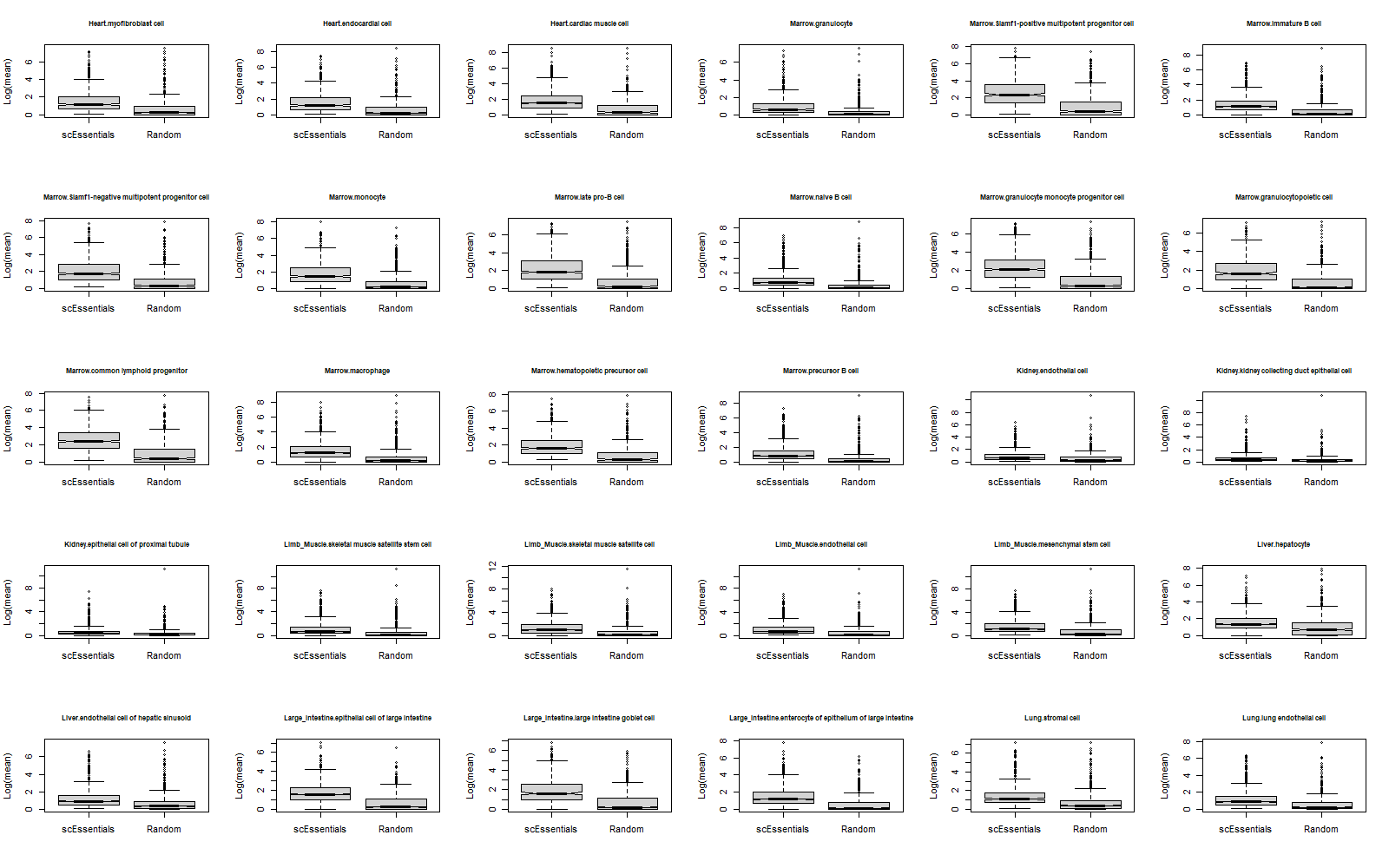


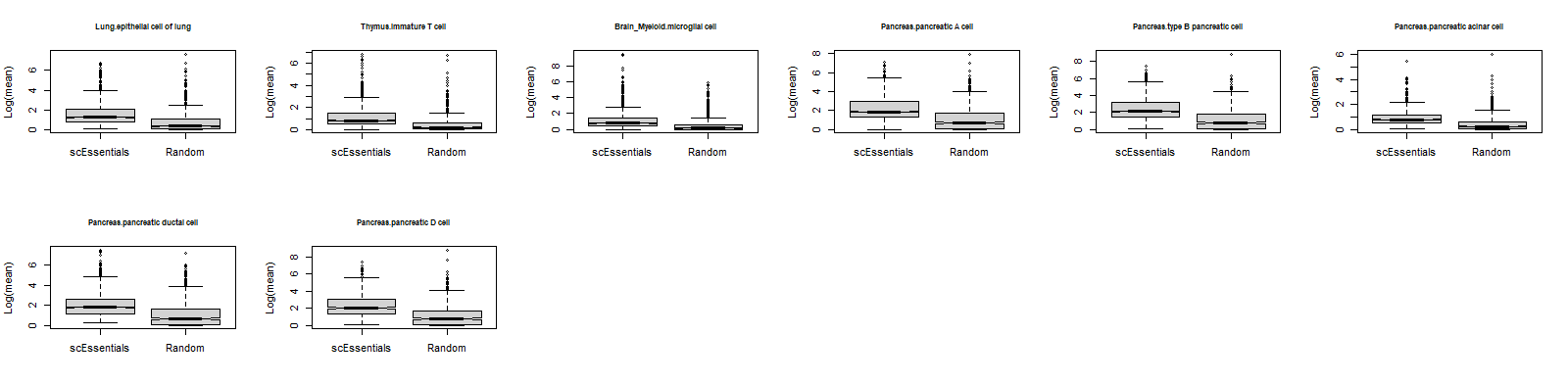


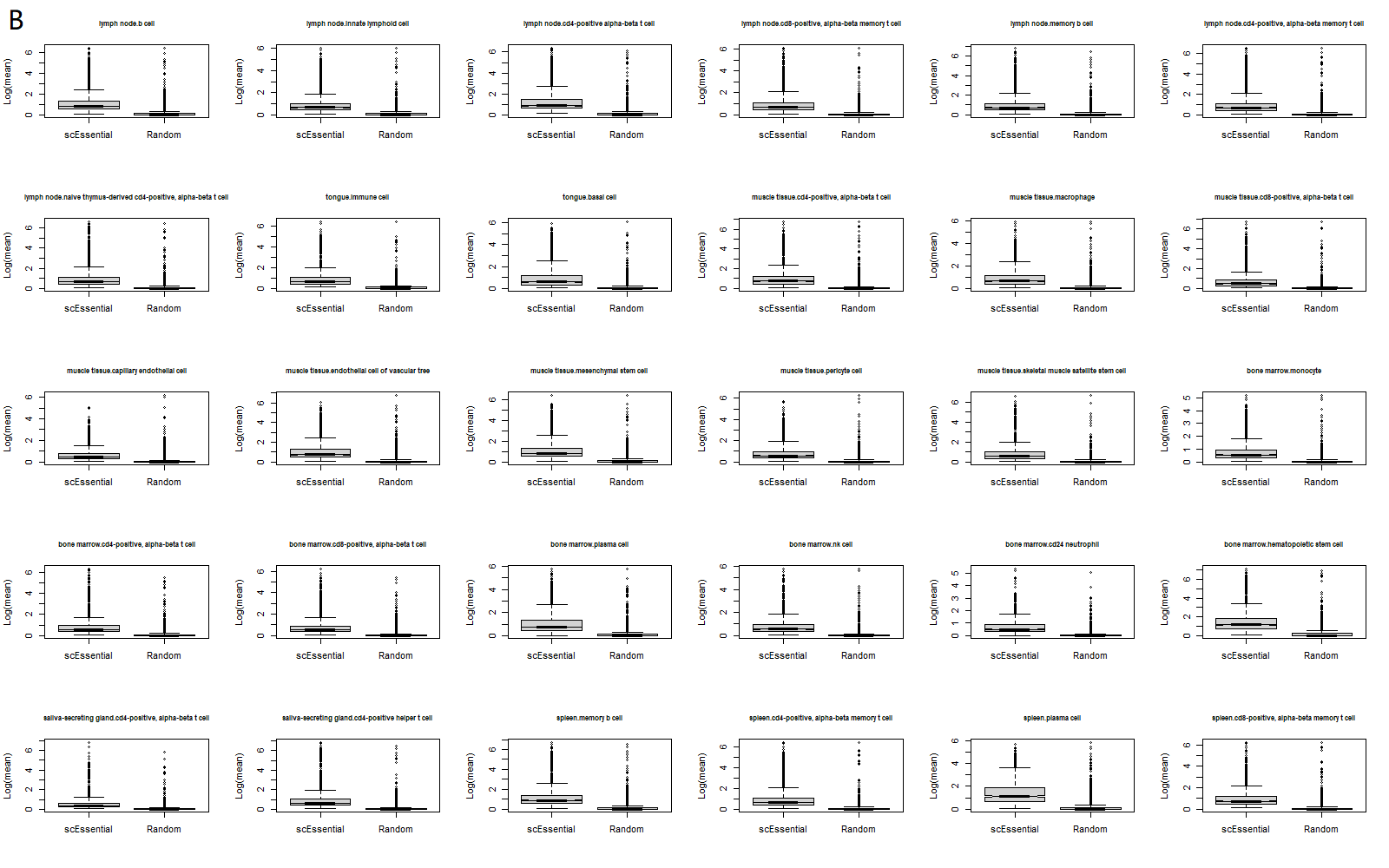


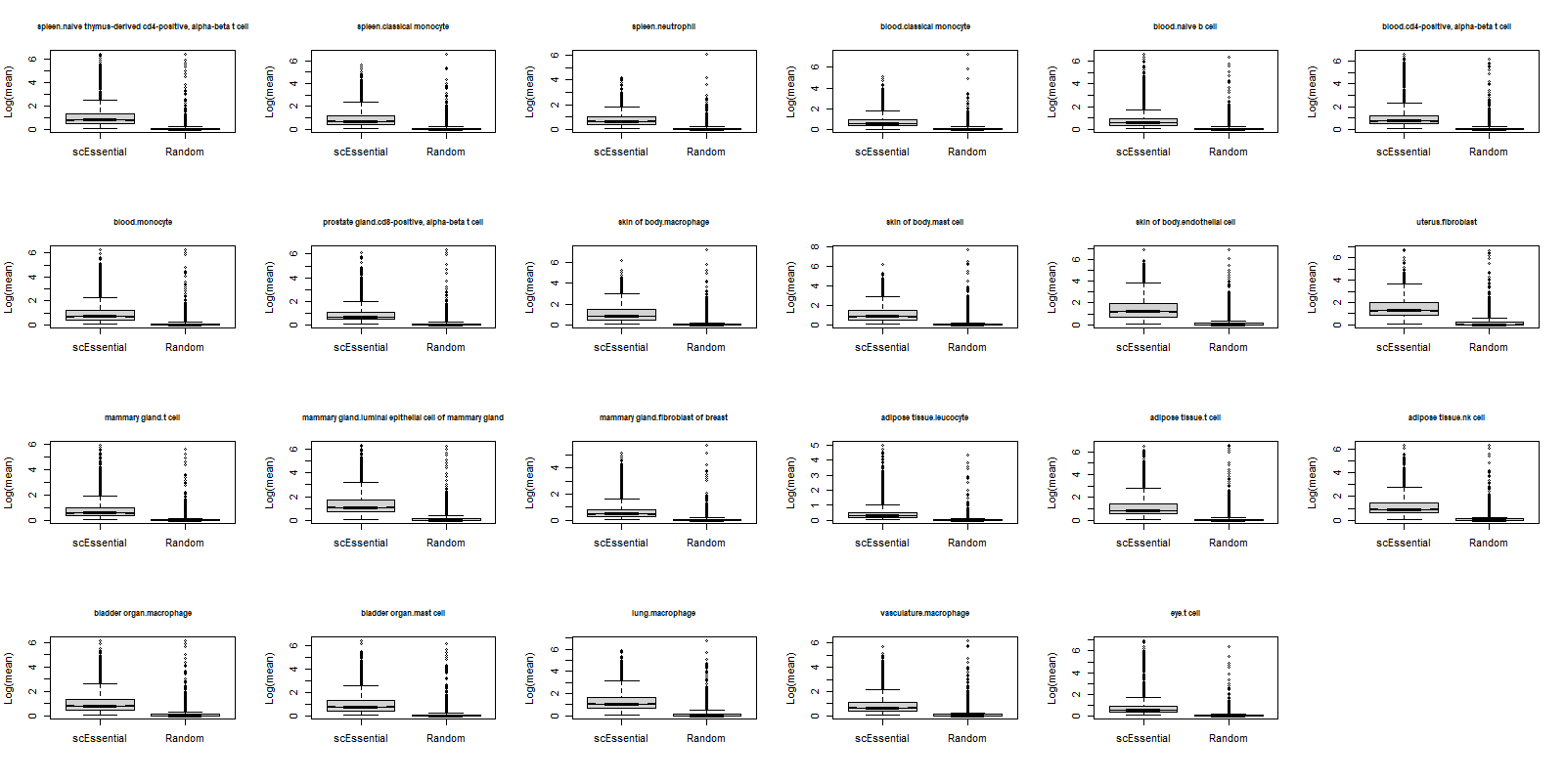


Supplementary Figure 8. The significantly high average expression in scEssentials as compared to one of the ten random gene lists. The Wilcoxon ranked test was applied for all cell types to compare the log of the mean expression difference in A) TM and B) TS. All cell types under various comparisons showed a significant increase in scEssentials with respect to the random gene list (Wilcoxon rank test, P-value < 0.05).


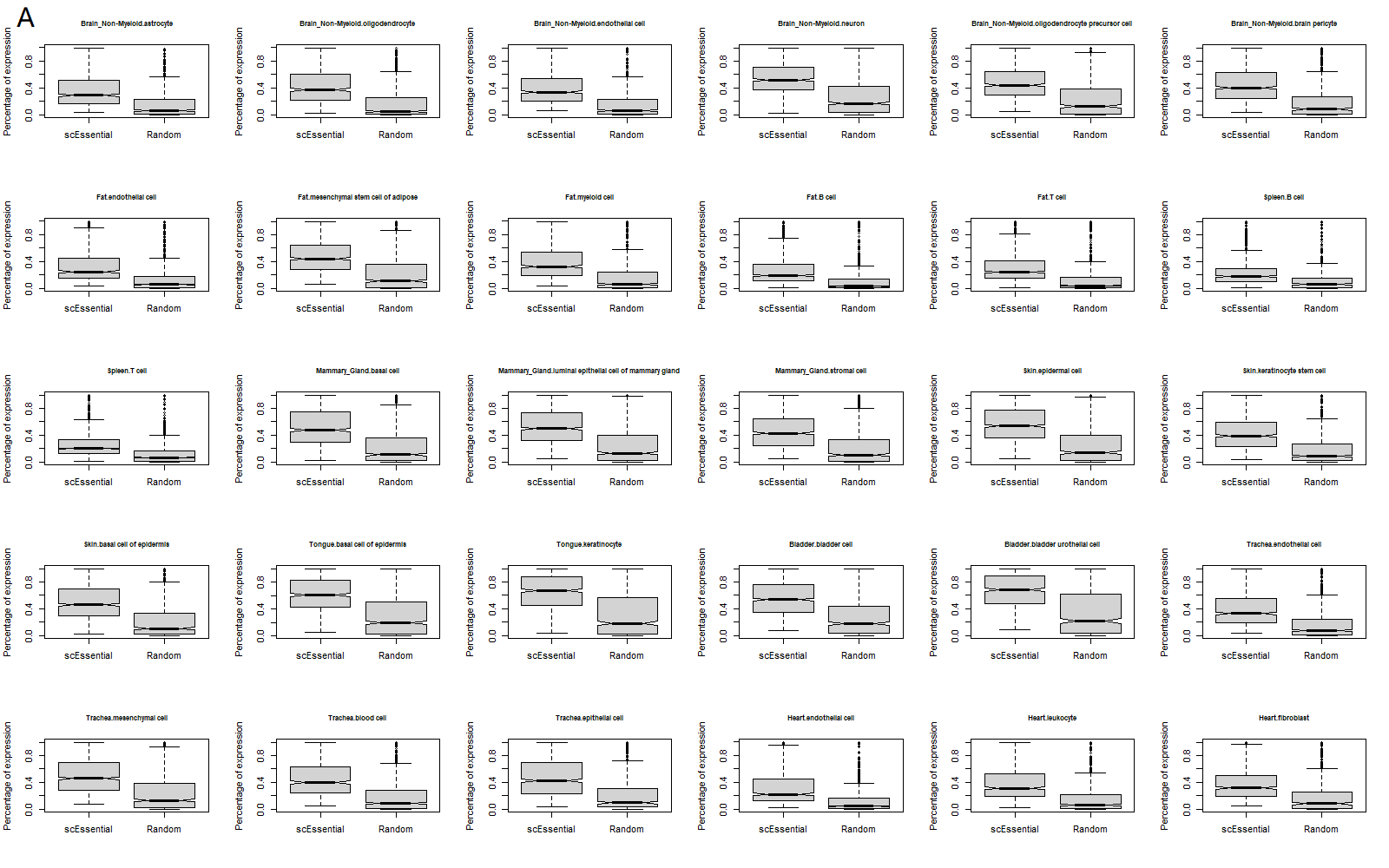


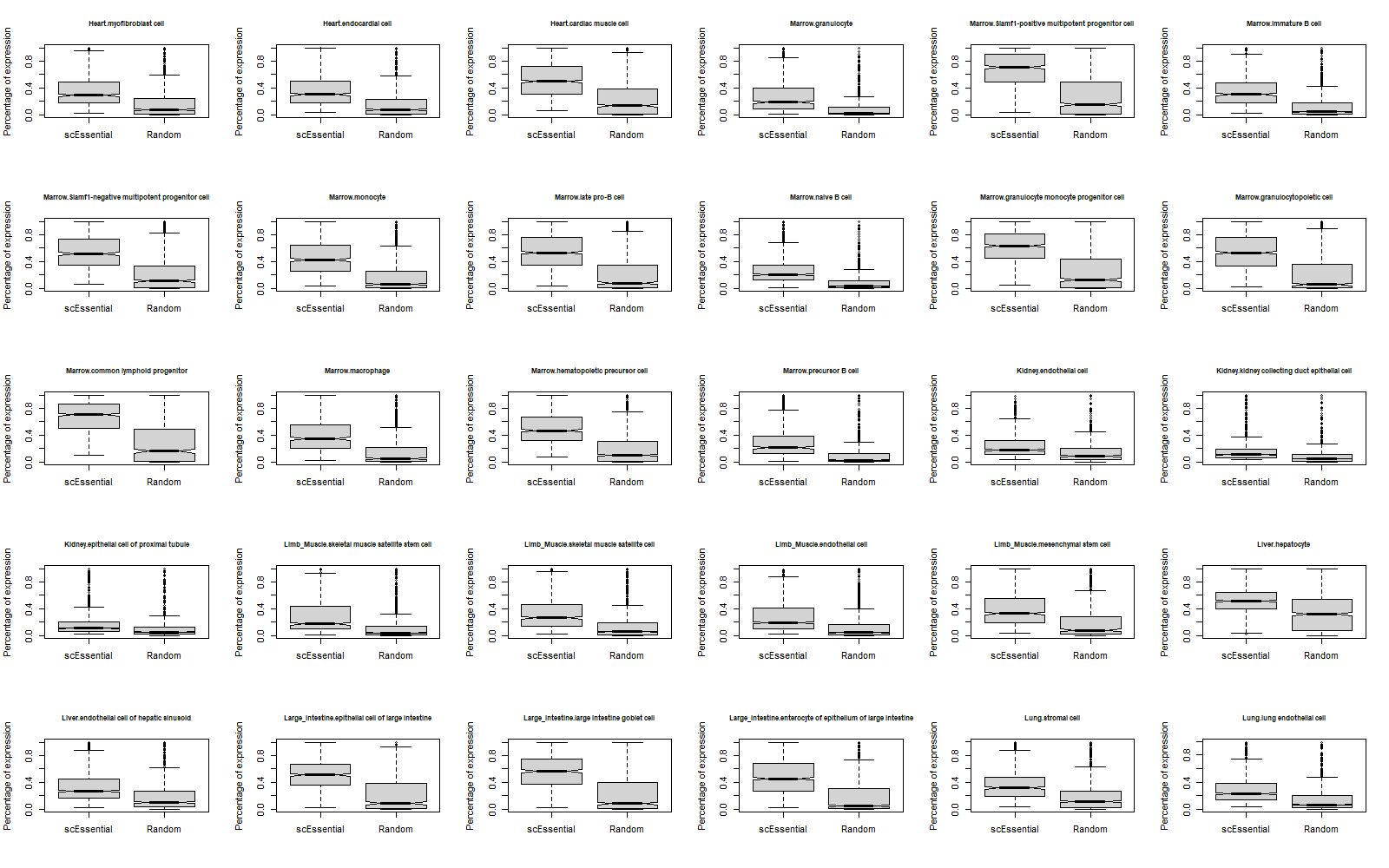

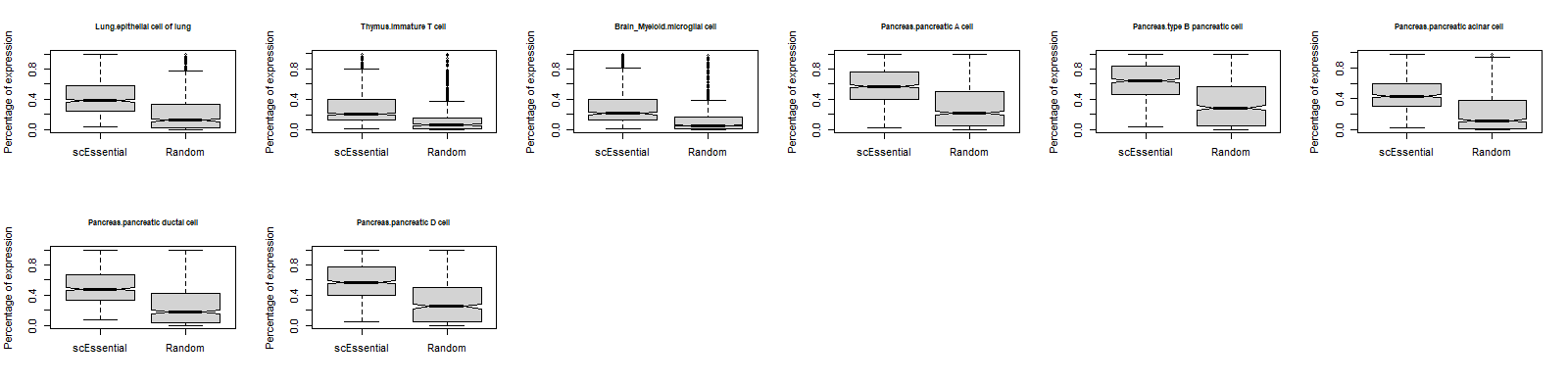


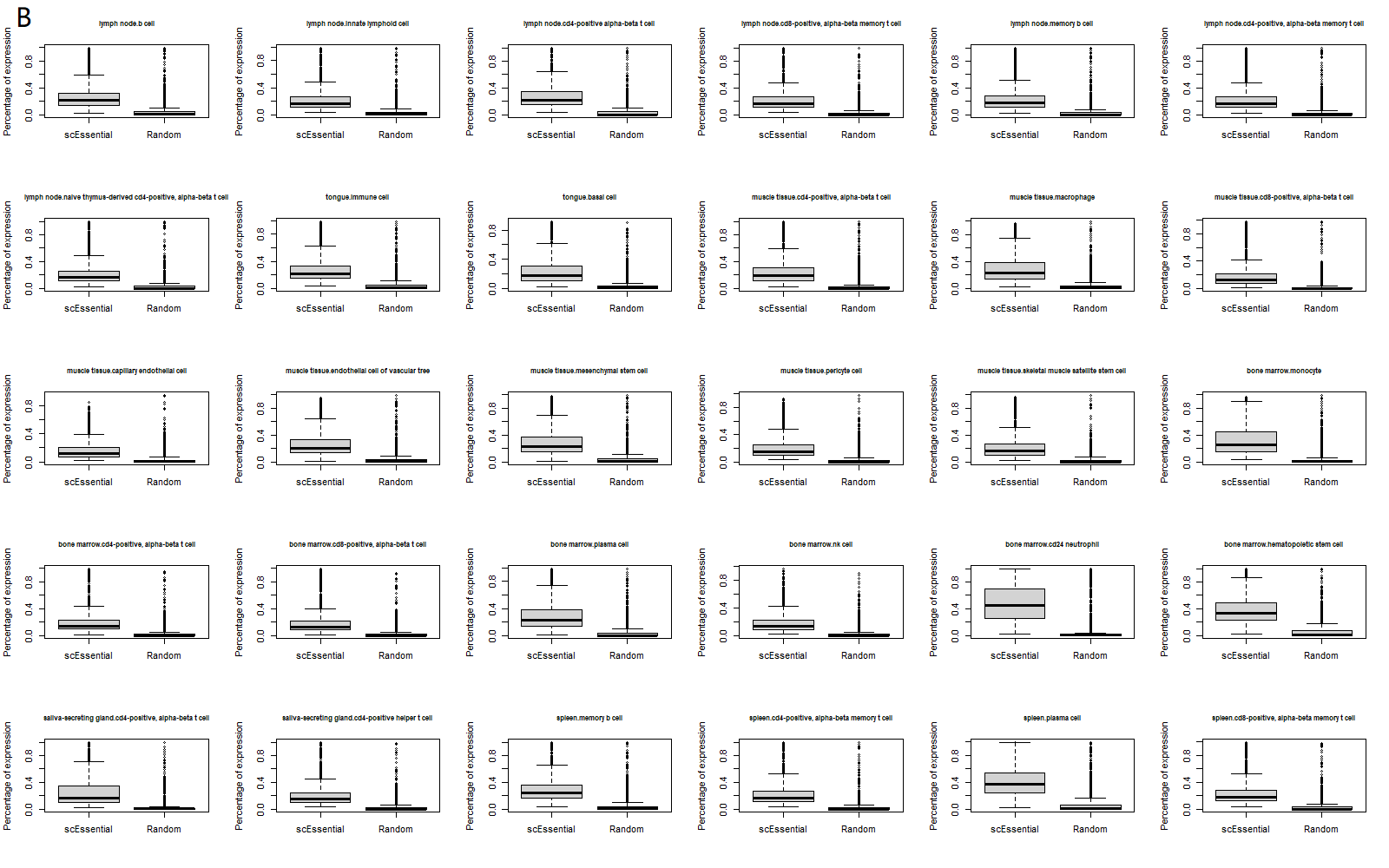


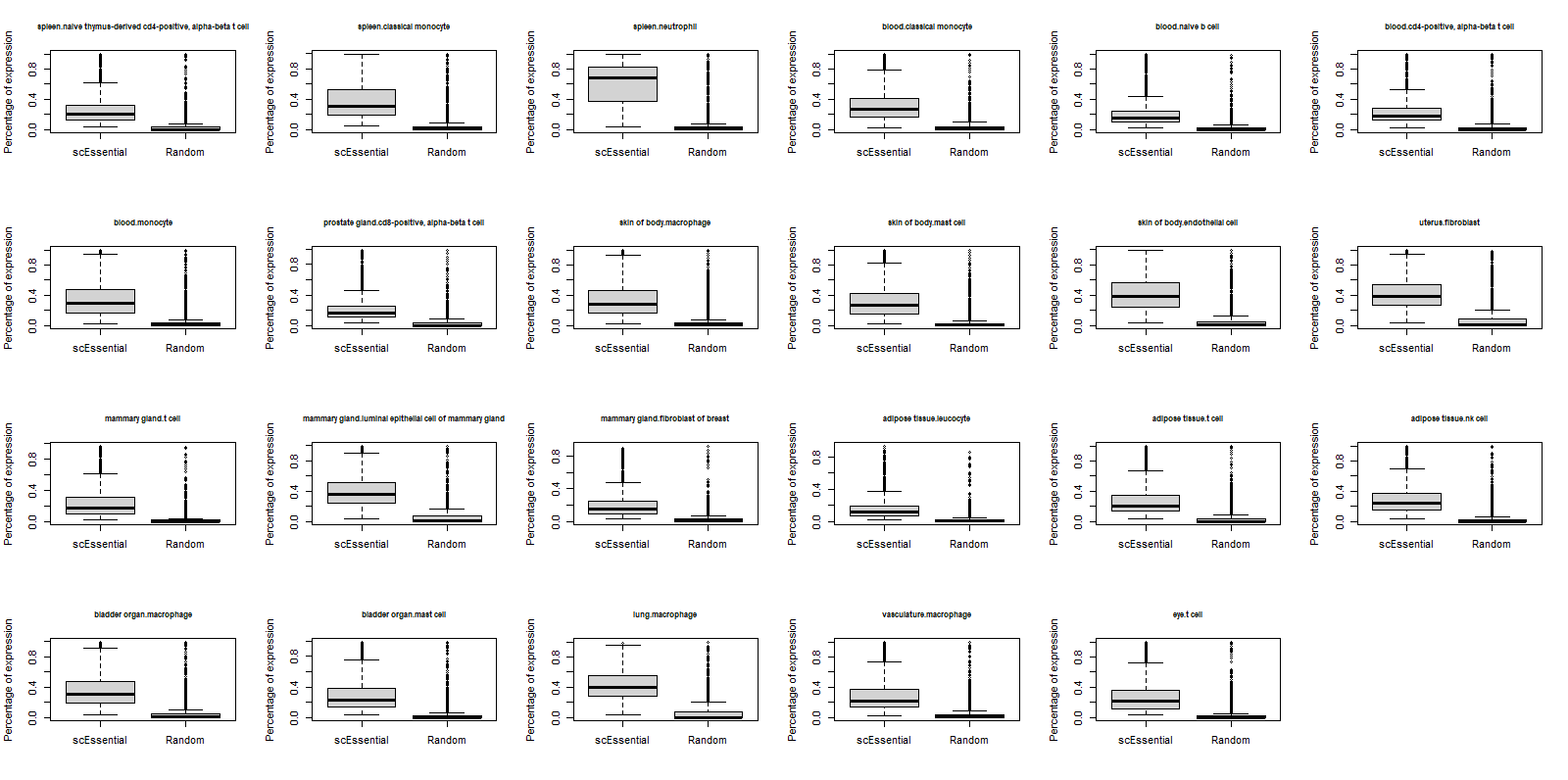


Supplementary Figure 9. The significantly high percentage of cells expressed in scEssentials genes as compared to one of the ten random gene list. The Wilcoxon ranked test was applied for all cell types to compare the percentage of expression difference in A) TM and B) TS. All cell types under various comparisons showed a significant increase in scEssentials genes with respect to the random gene list (Wilcoxon rank test, P-value < 0.05).


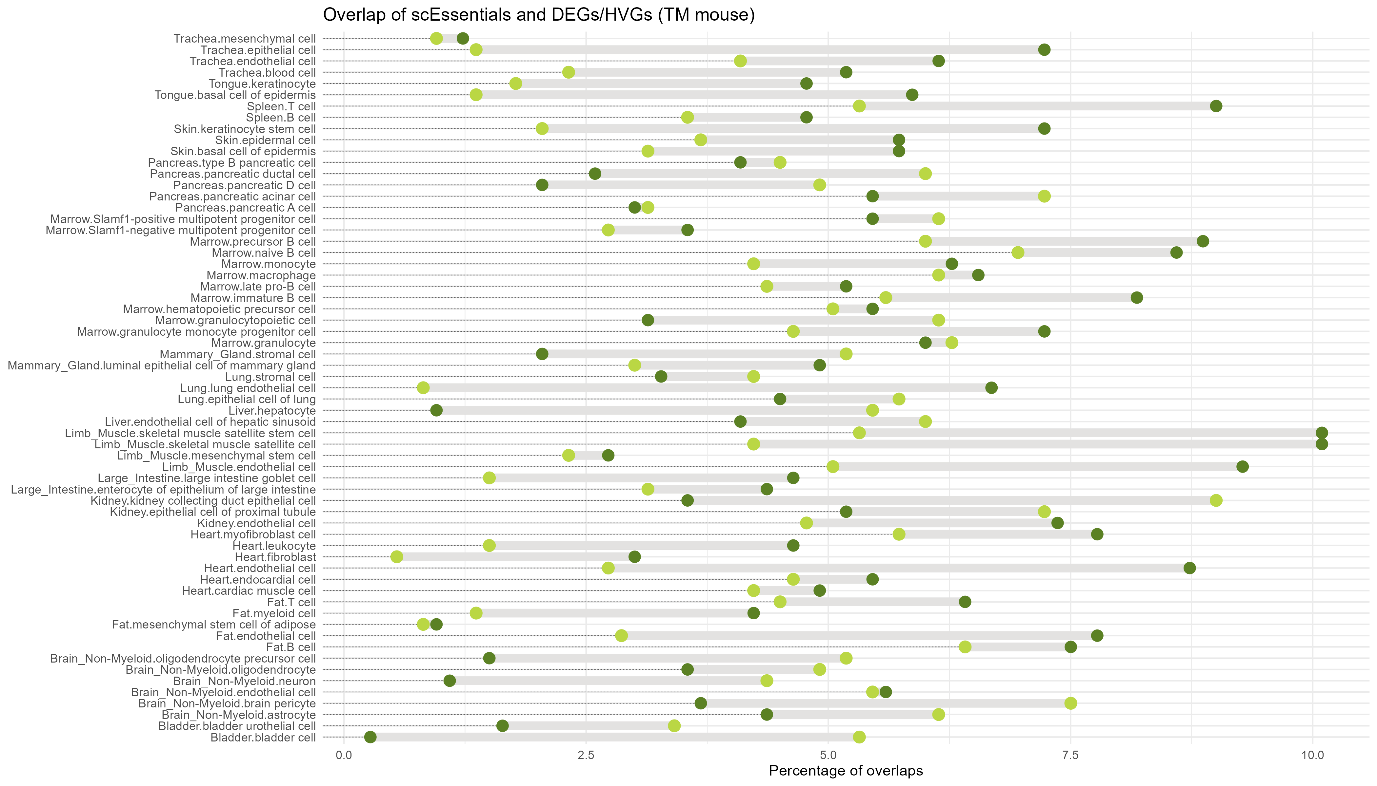


Supplementary Figure 10. Investigating the degree of overlap between scEssentials genes and two types of gene sets, cell-type specific differentially expressed markers (DEGs) and highly variable genes (HVGs) for cell types in TM (mouse). Dark green represented the overlap with DEGs and light green represented the overlap with HVGs.


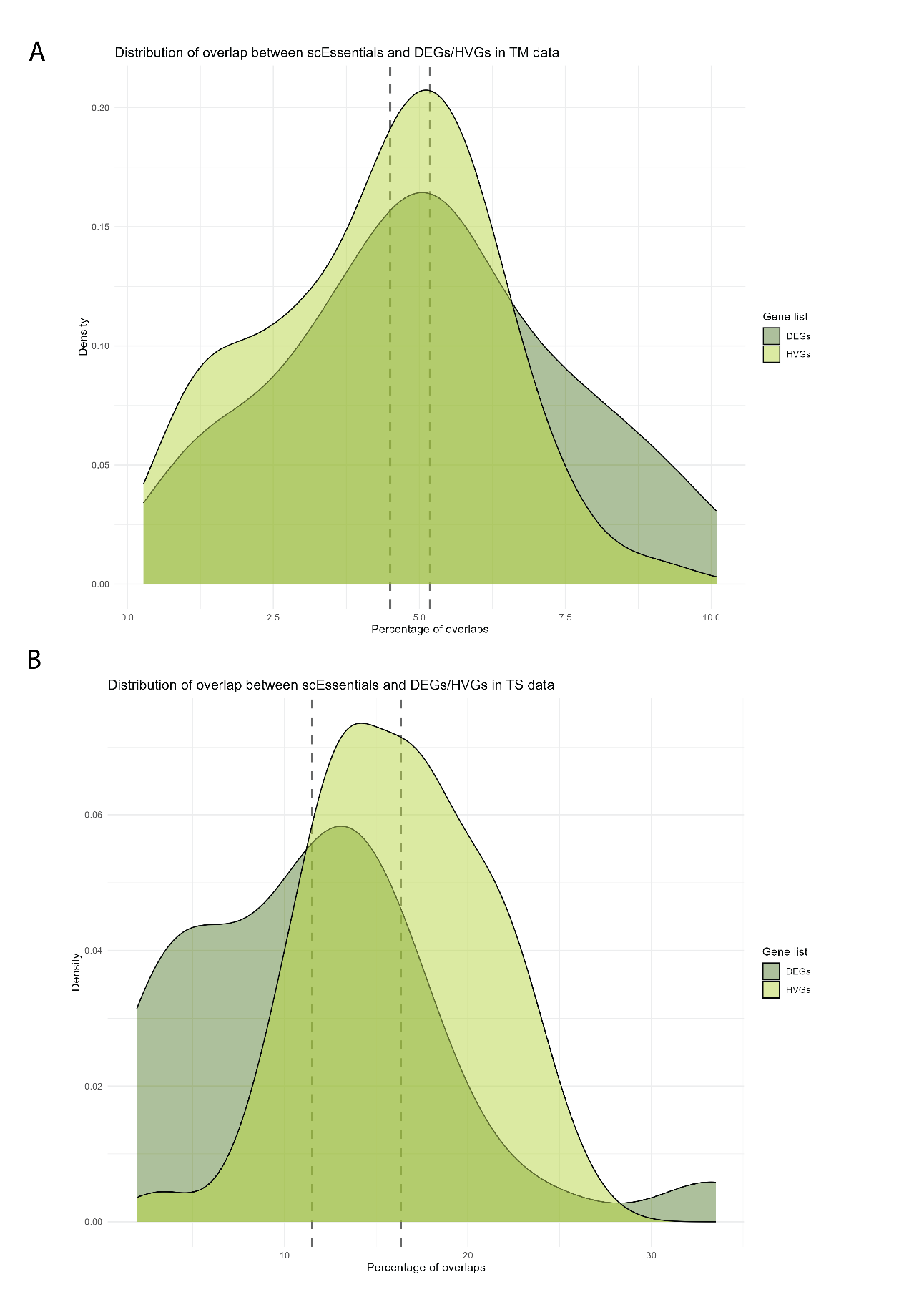
Supplementary Figure 11. Density plot showed the distribution of the percentage of overlaps between scEssentials genes and DEGs/HVGs across all cell types in A) TM (mouse) and B) TS (human). Dark green represented the overlap with DEGs and light green represented the overlap with HVGs. Dash line indicated the median value.


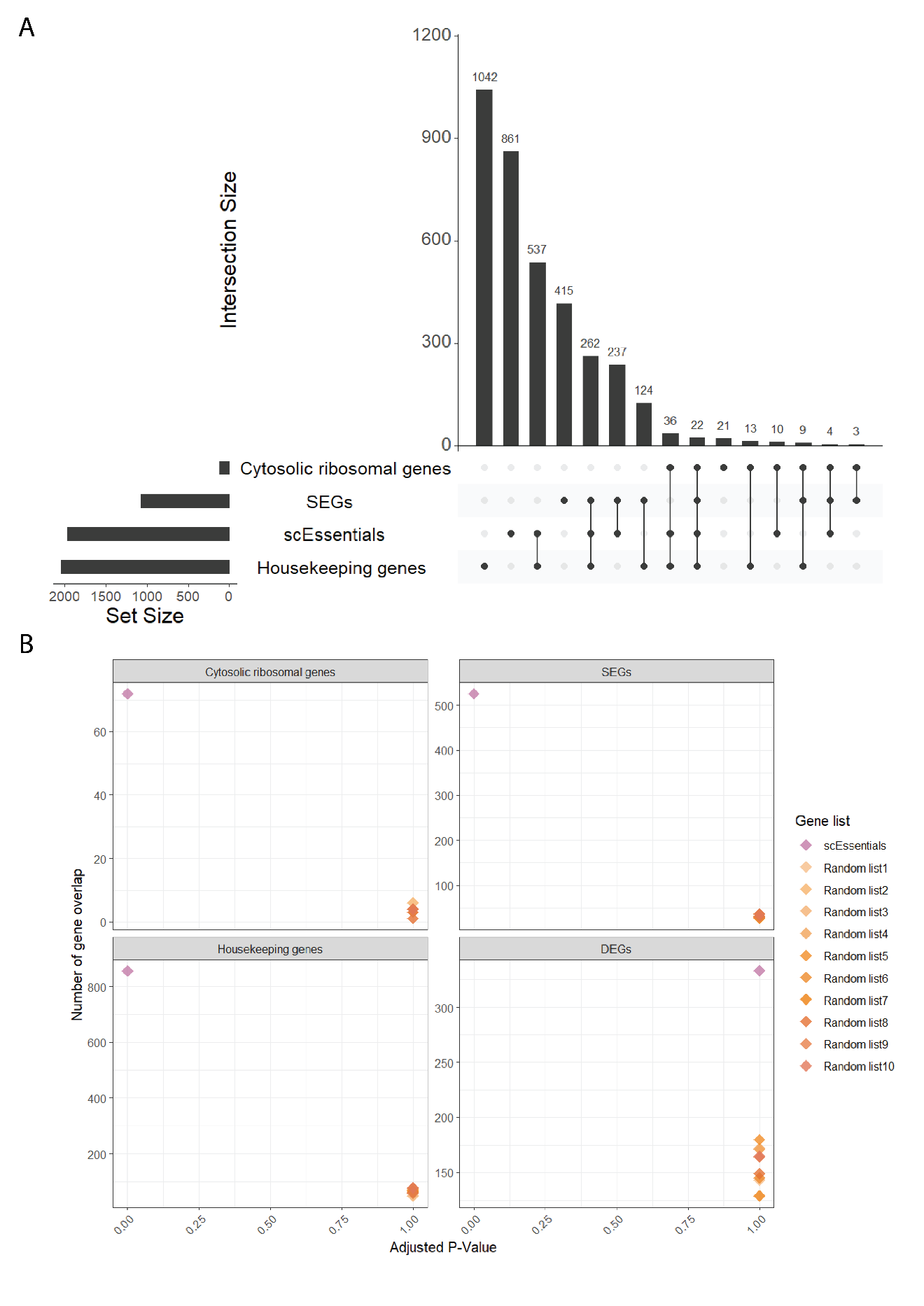


Supplementary Figure 12. Assessing the overlap between scEssentials genes and specific gene sets (5-7). A) An upset plot demonstrated the overlap between scEssentials genes and gene sets that represent ribosomal genes, stably expressed genes (SEGs), and housekeeping genes . B) A dotplot demonstrated the statistical significance of these overlaps using a hypergeometric test for scEssentials genes relative to 10 random gene lists. scEssential genes overlapped significantly with ribosomal genes, SEGs, and housekeeping genes but not DEGs whereas all random gene lists had overlaps with these 4 gene lists that were demonstrably not statistically significant (adjusted P-value < 0.05).


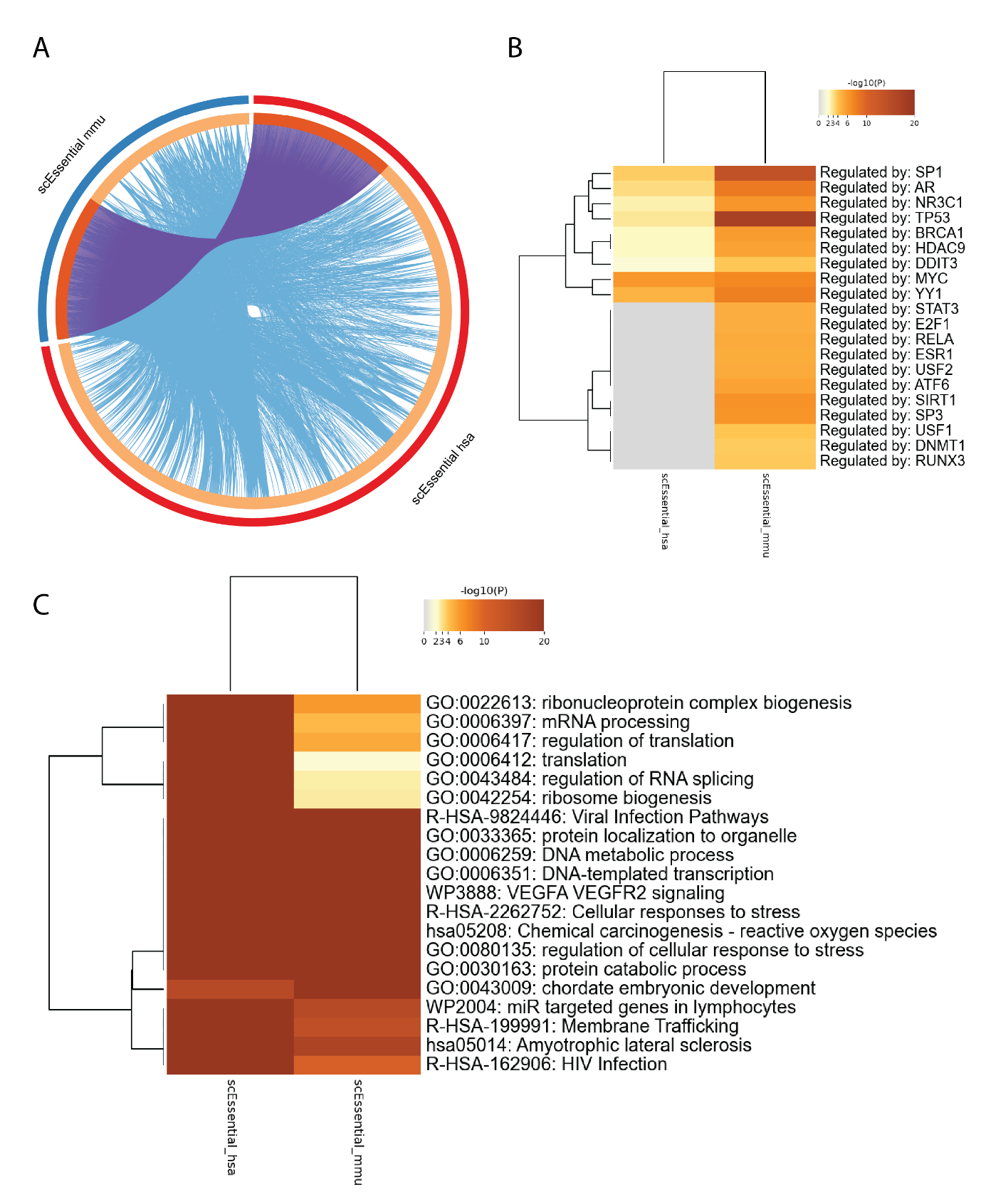


Supplementary Figure 13. Characterisation of human and mouse scEssentials from Metascape. A) Circos plot compared the paralogs between two gene lists. B) Enrichment of transcription factors in each gene list. C)Top 20 enriched biological pathways in different databases.


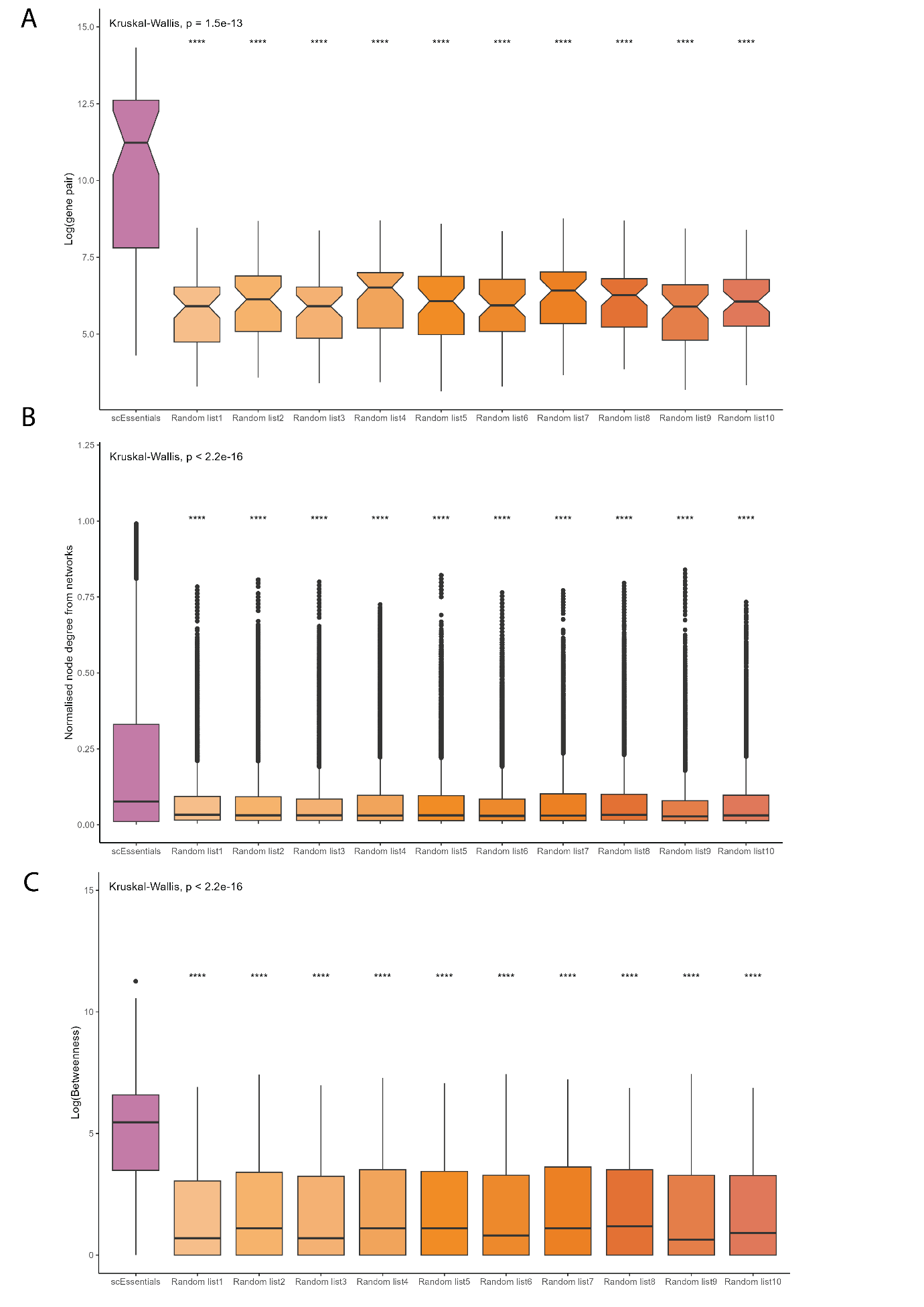


Supplementary Figure 14. Analysing the correlation of human scEssentials gene pairs against 10 random gene lists. Boxplots illustrated A) the number of significant gene pairs; B) the network betweenness; and C) the network normalised node degrees that are constructed from scEssentials and random gene lists. (P-value *<0.05, ** <0.01, ***, <0.001, **** 0.0001).


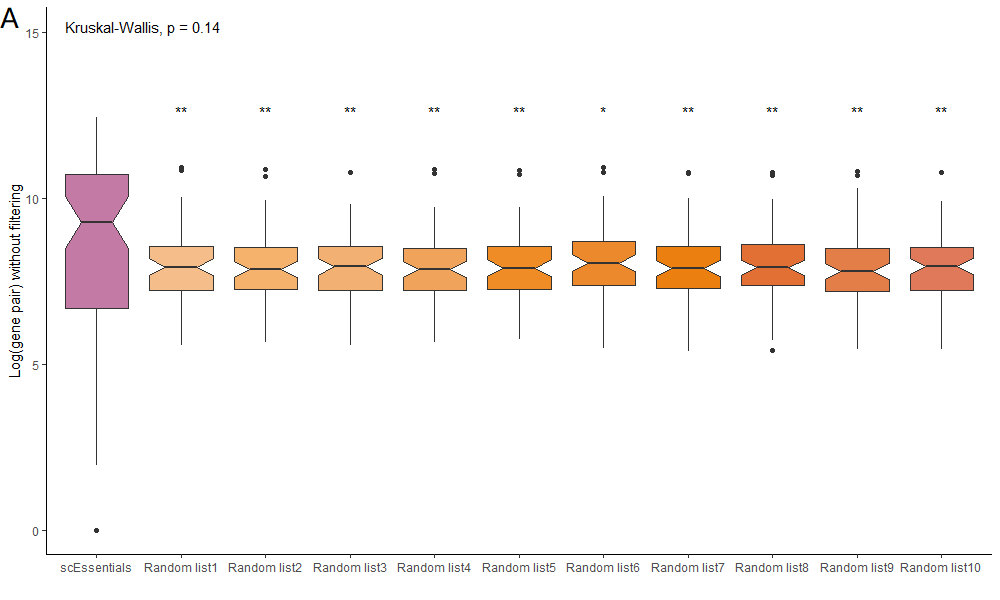

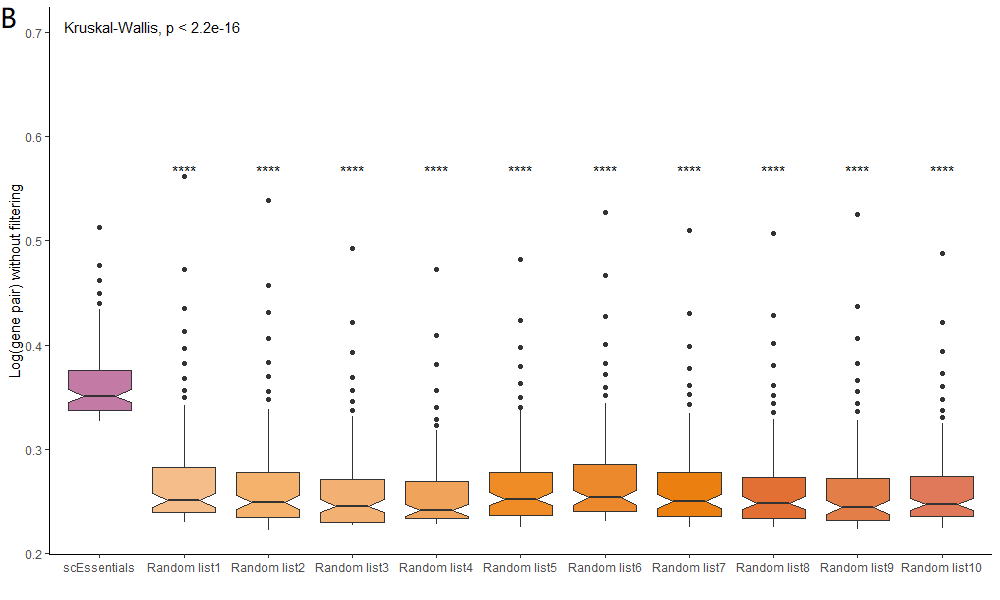


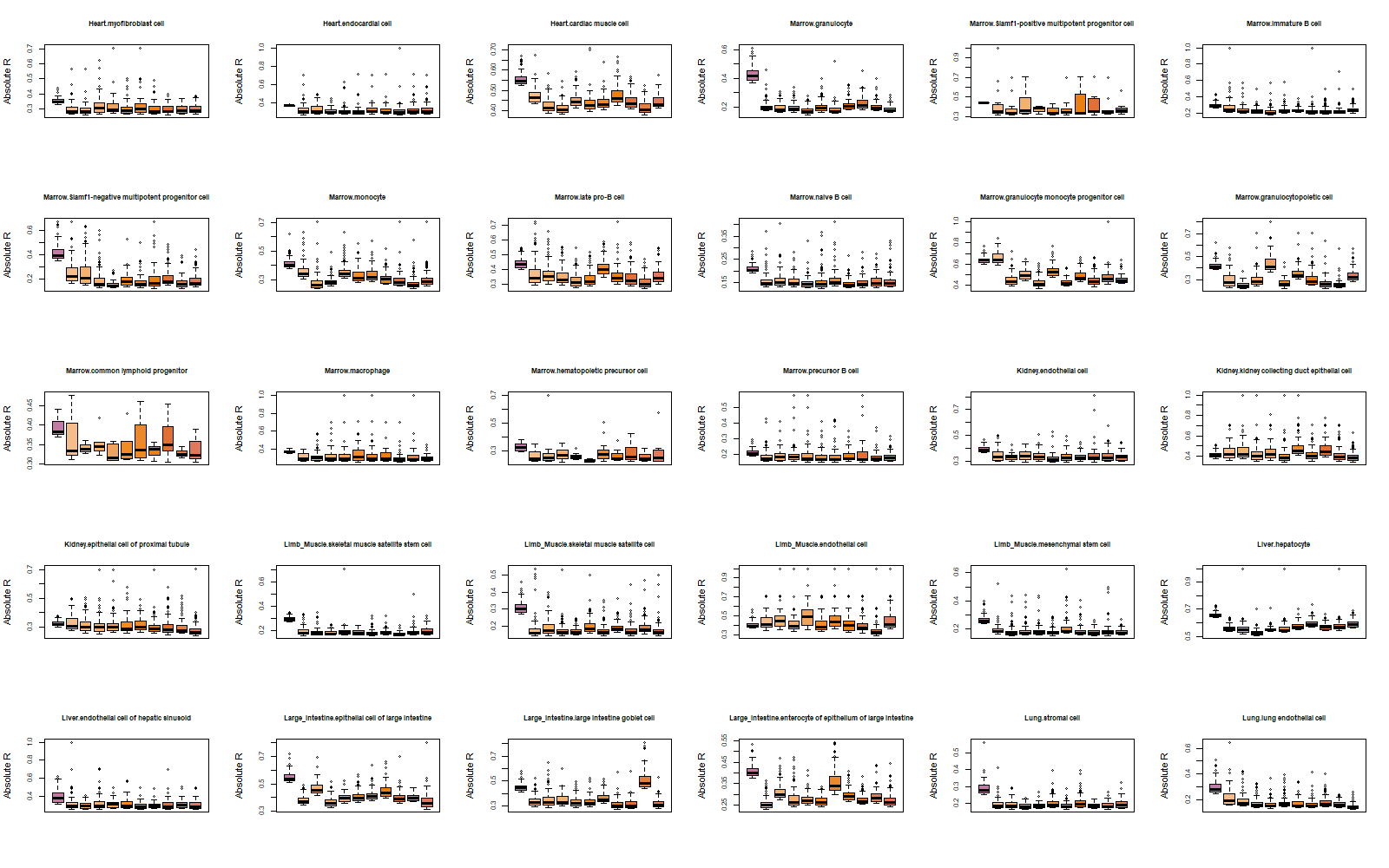

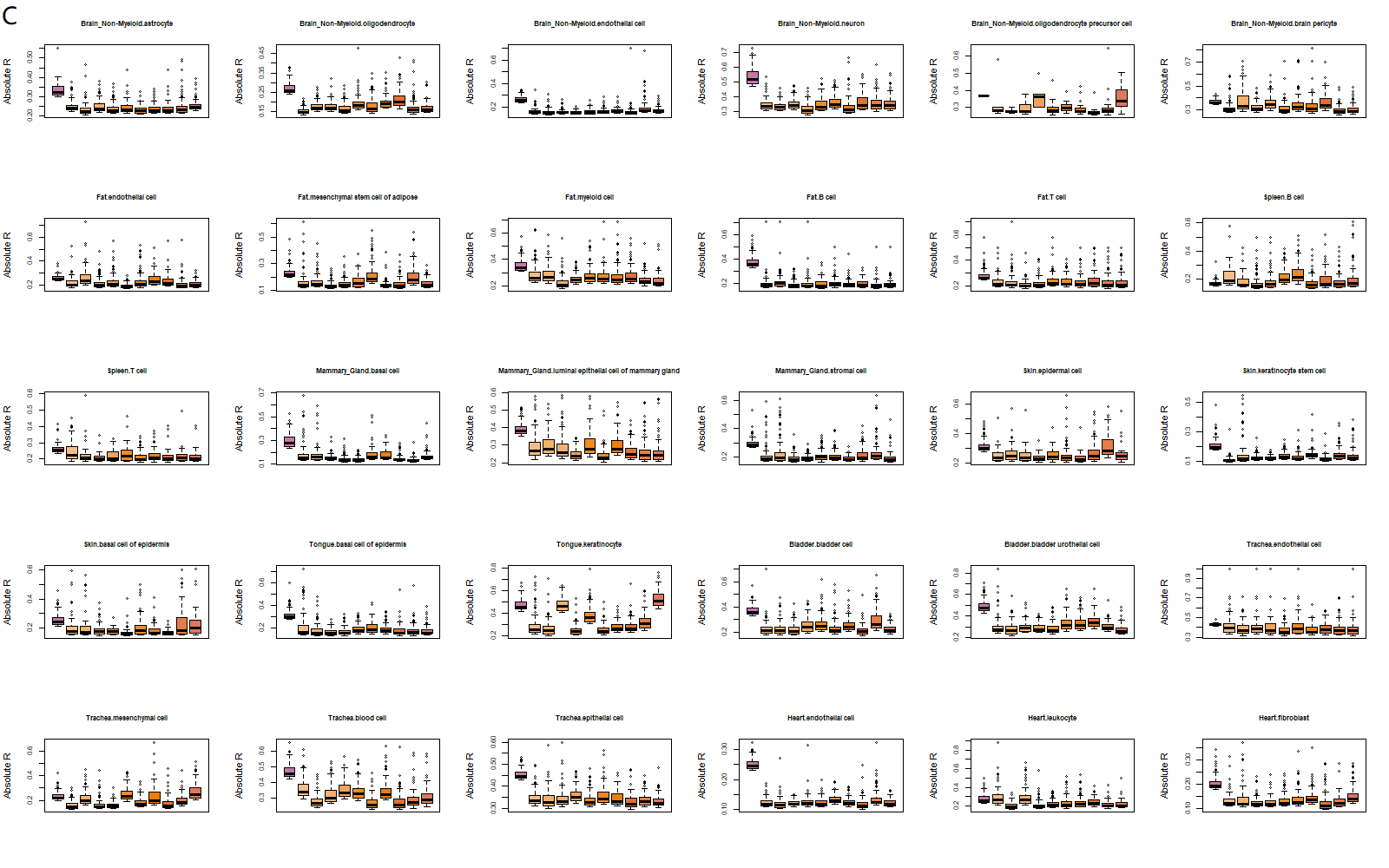


Supplementary Figure 15. Coexpression level of mouse scEssentials gene compared to 10 randomly sampled gene lists. A) The number of significantly correlated gene pairs for scEssentials genes as compared to other 10 random gene lists. Boxplots for all the genes in each gene list without filtering. B) The absolute correlation of coefficient for the top 100 most significantly coexpressed gene pairs for scEssentials as compared to the other 10 random gene lists. Boxplots for all the genes in each gene list after filtering. *Kruskal-Wallis* test was applied to determine the significance (* p<0.05; ** p<0.01; *** p<0.001. **** p<0.0001). C) The absolute correlation of coefficient for the top 100 most significantly coexpressed gene pairs in 68 cell types. Notably, only Slamf1-positive multipotent progenitor cell and common lymphoid progenitor cells did not show significantly higher correlation efficient in scEssentials with respect to other randomly sampled gene lists.


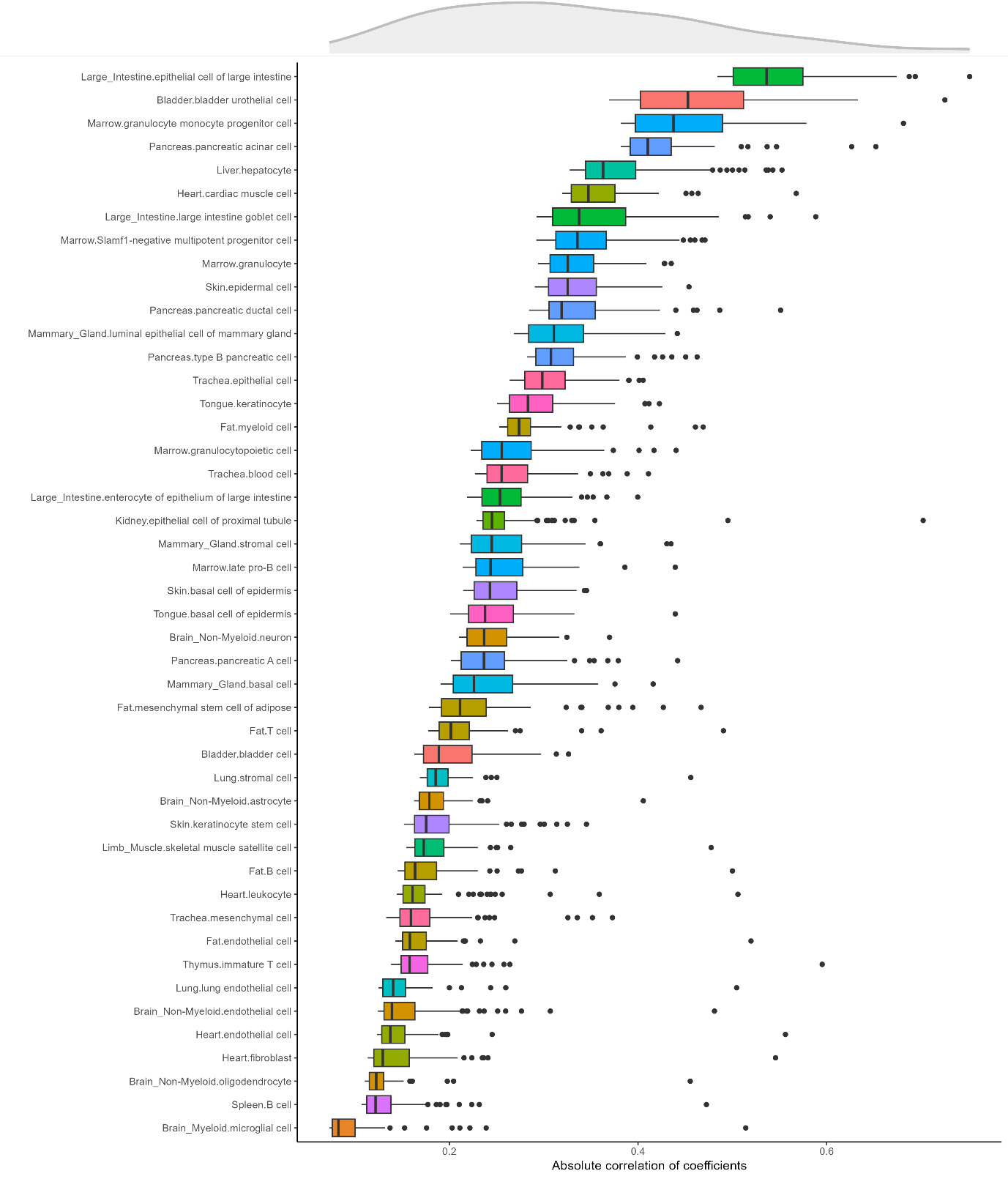


Supplementary Figure 16. Analysing the top 100 significantly correlated mouse ribosomal gene pairs across TM cell types (coloured by tissue origin). Median value of the correlation is 0.24


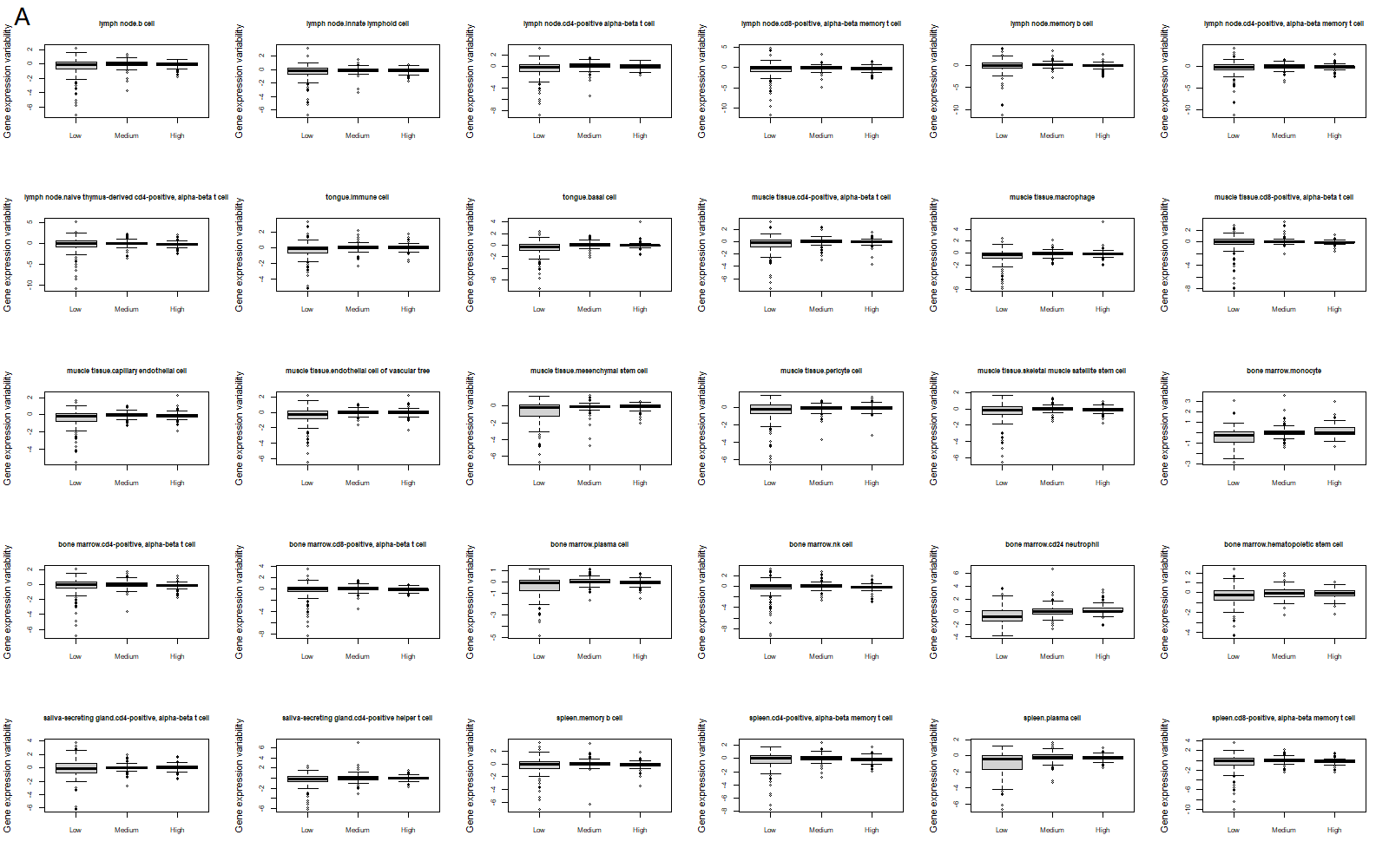

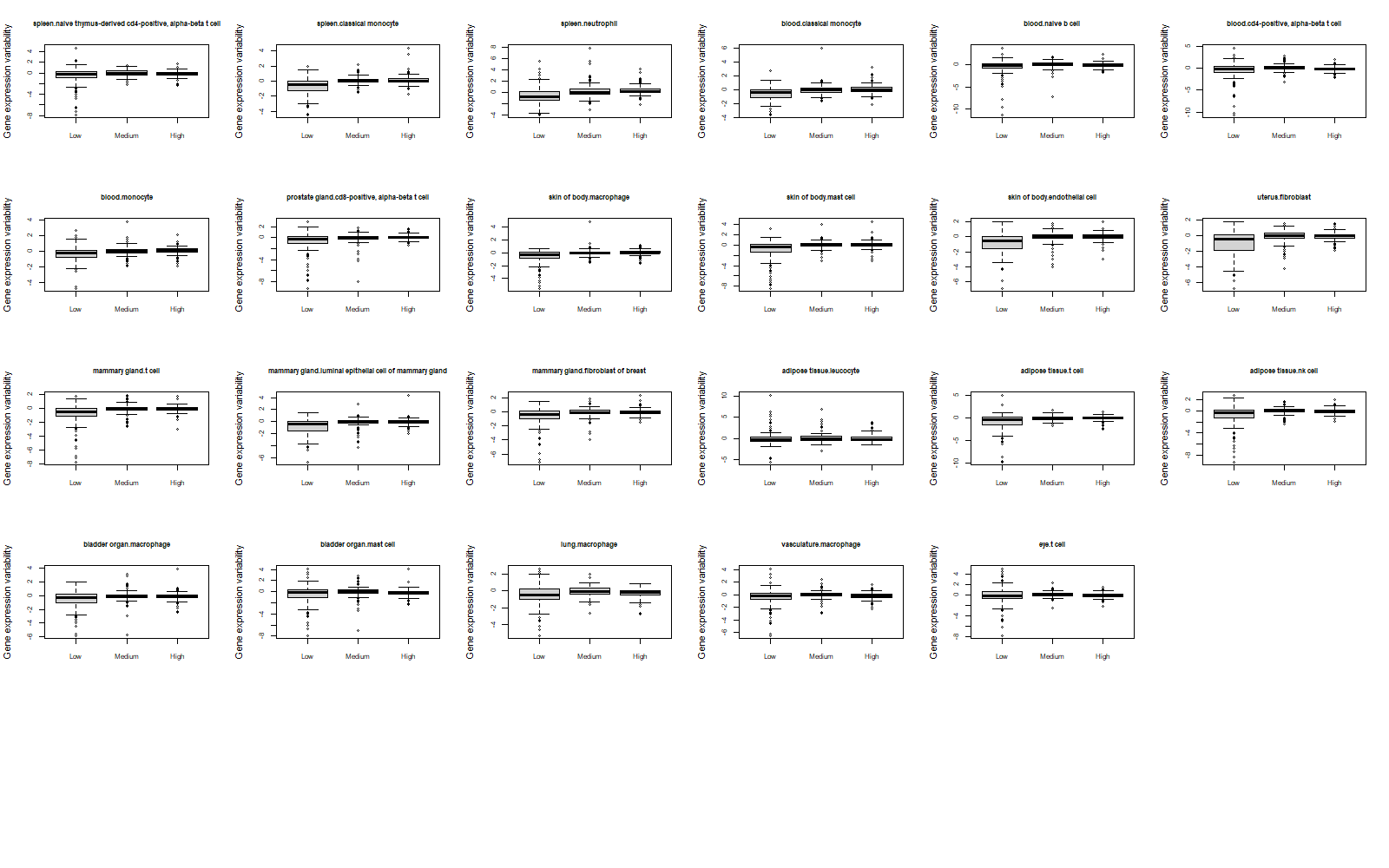


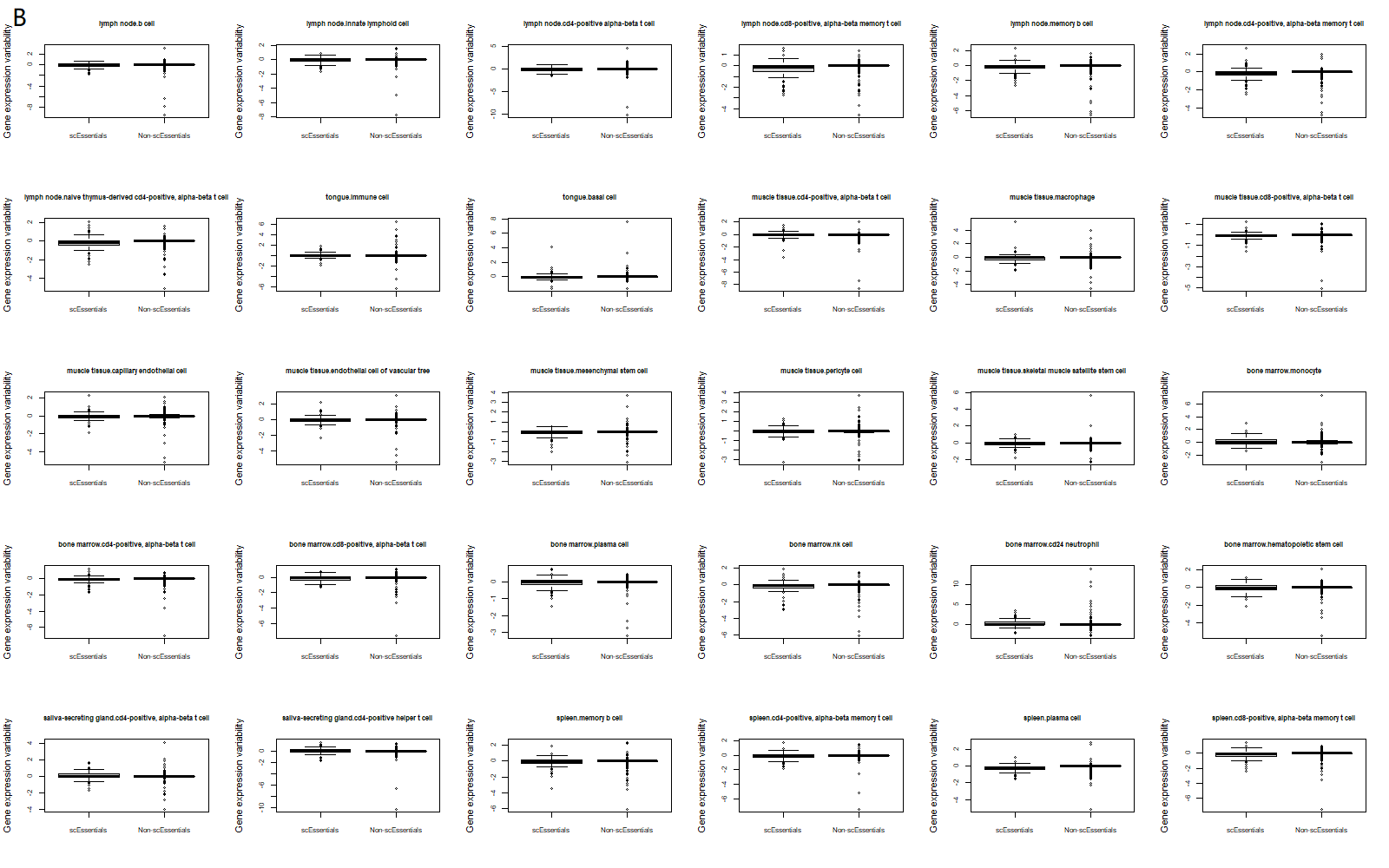

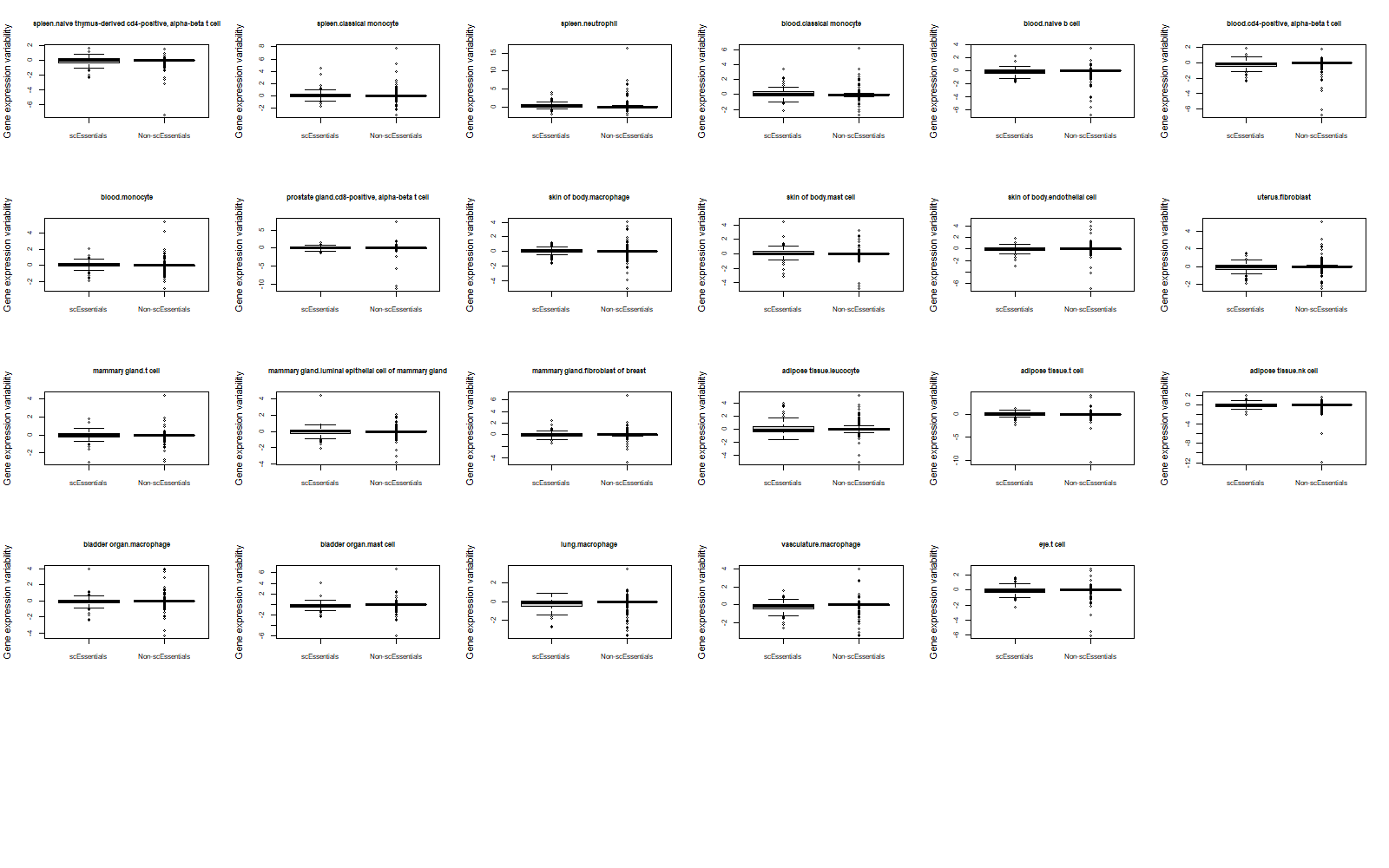


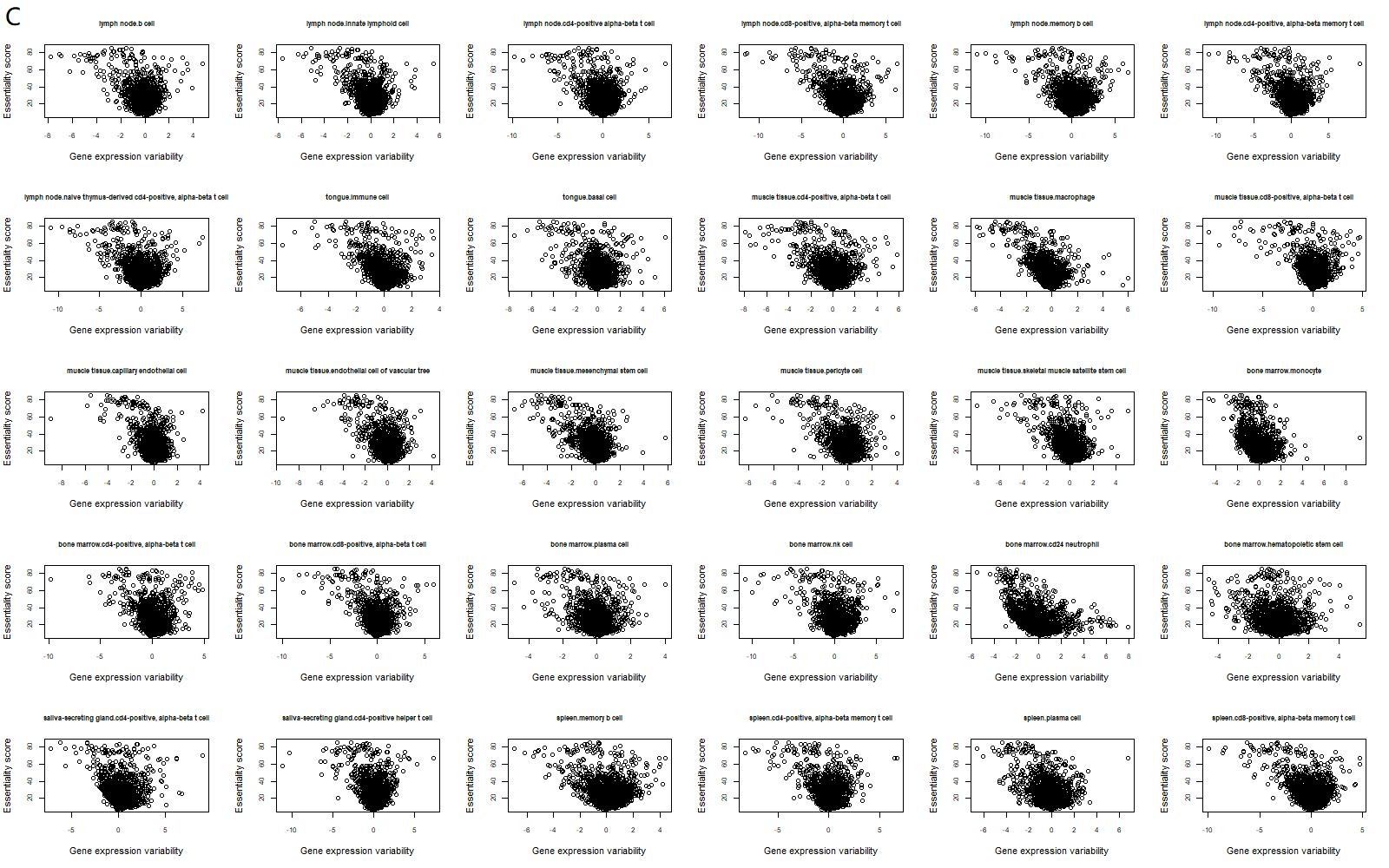

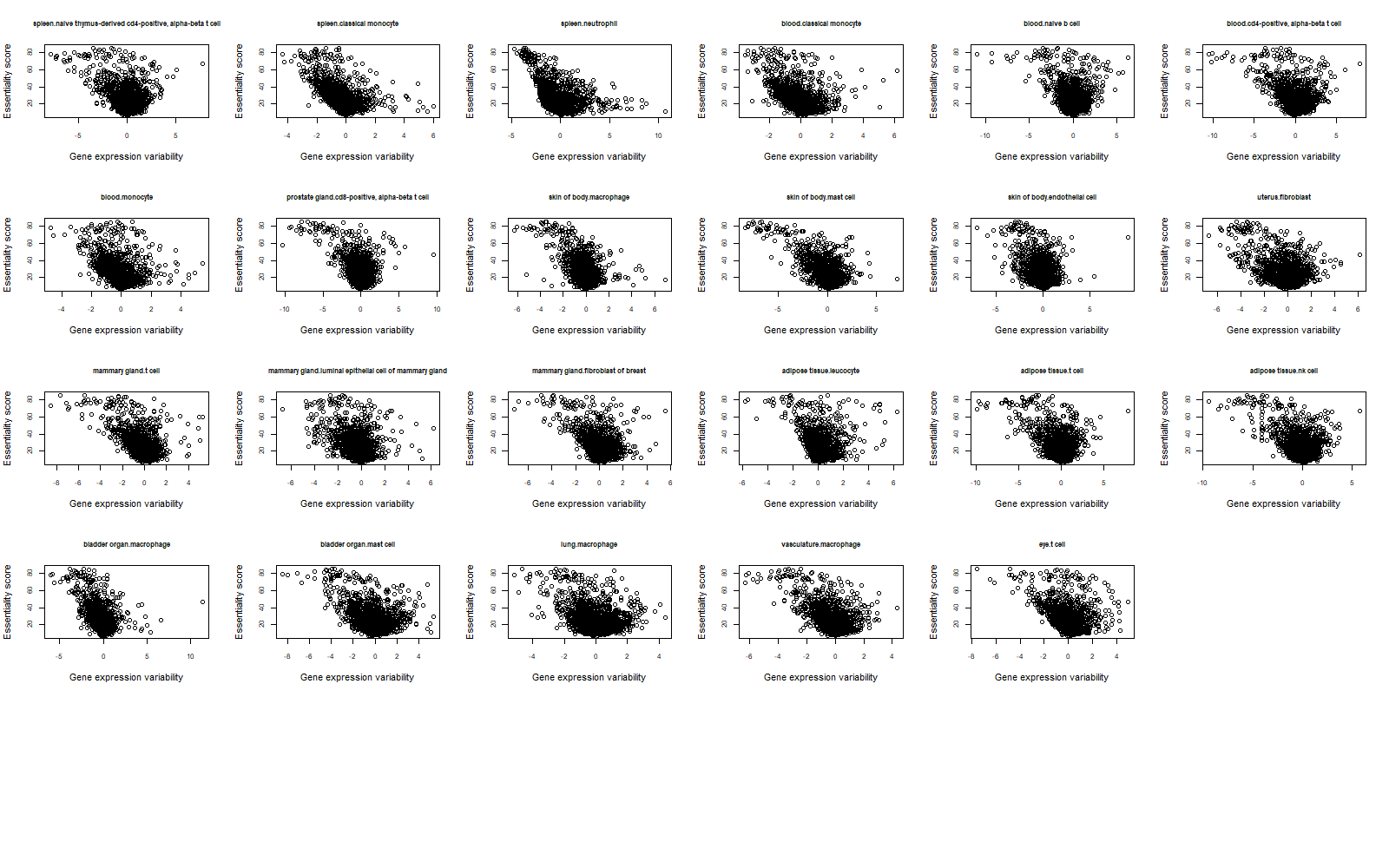
Supplementary Figure 17. Relationship of scEssential genes with different gene damage index (8). A) Boxplot for gene expression variability for each cell type under low, medium and high risks in each cell type. B) Boxplot for gene expression variability for each cell type between scEssentials and non-scEssentials under high risks. C) Correlation between ES and scEssentials expression variability across all cell types. The correlation coefficients were measured by Spearman correlation and were significantly correlated. The median correlation coefficient across all cell types was -0.4.


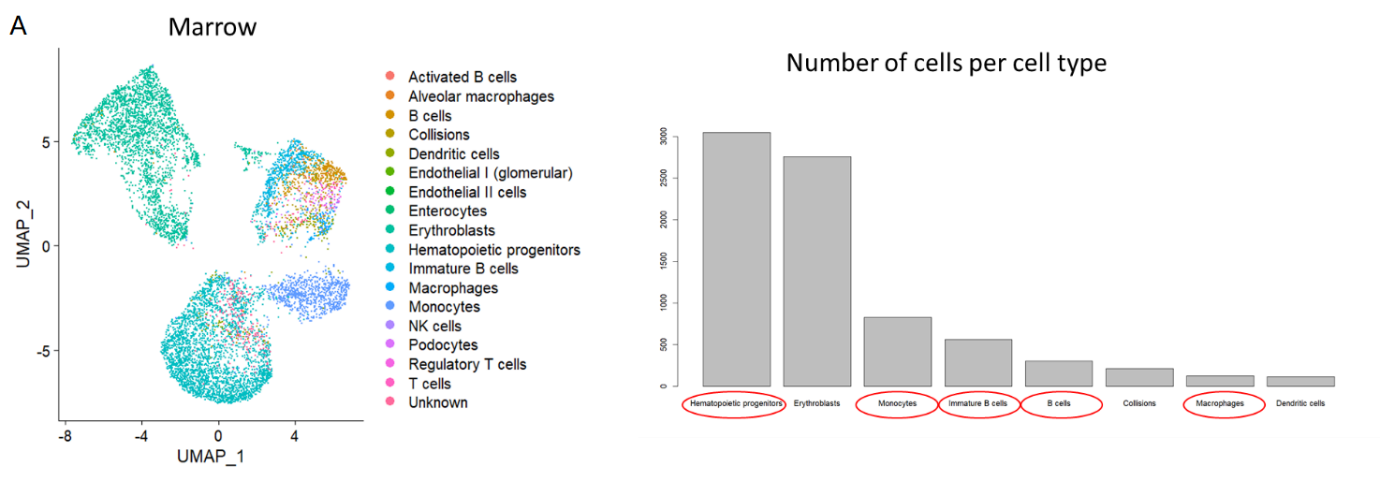


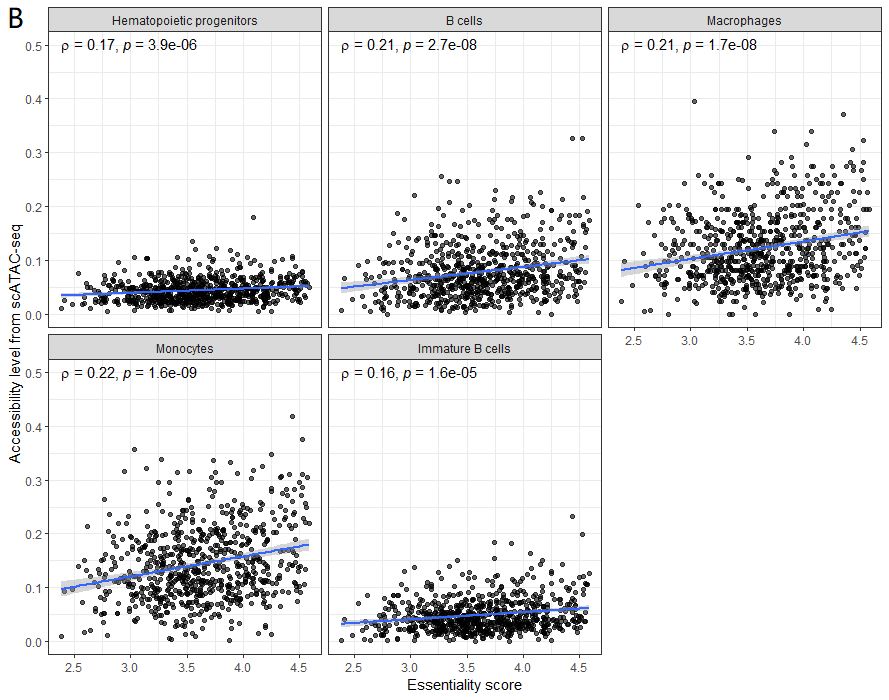


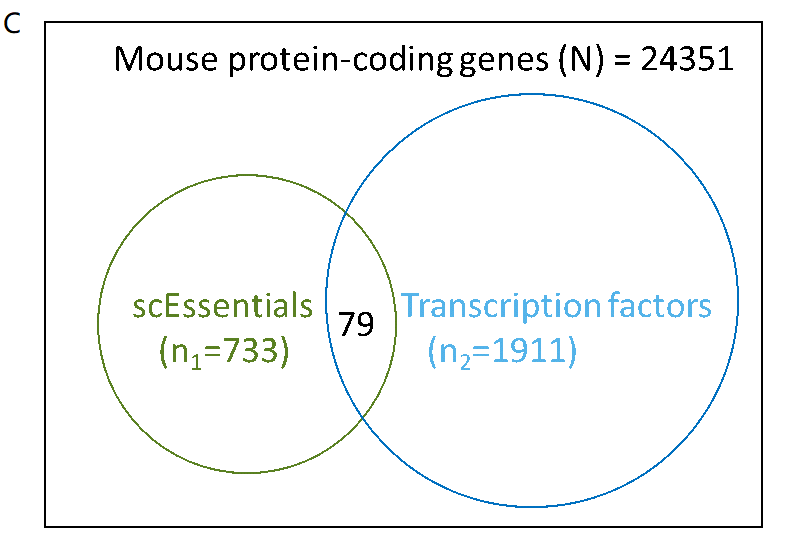


Supplementary Figure 18. Chromatin accessibility inferred from scATAC-seq mouse atlas (12). A) UMAP demonstrated the clustering of the cell types in bone marrow tissue based on scATAC-seq data, and the cell types with more than 100 cells were plotted as barplot where the cell type that overlapped with TM data was circled. B) Dotplot demonstrated the correlation between the essentiality score and the accessibility level for each cell type and on average. Spearman correlation was applied to measure the correlation. Blue line represented the linear regression between two variables. C) Illustration of the parameters used for the hypergeometric test.


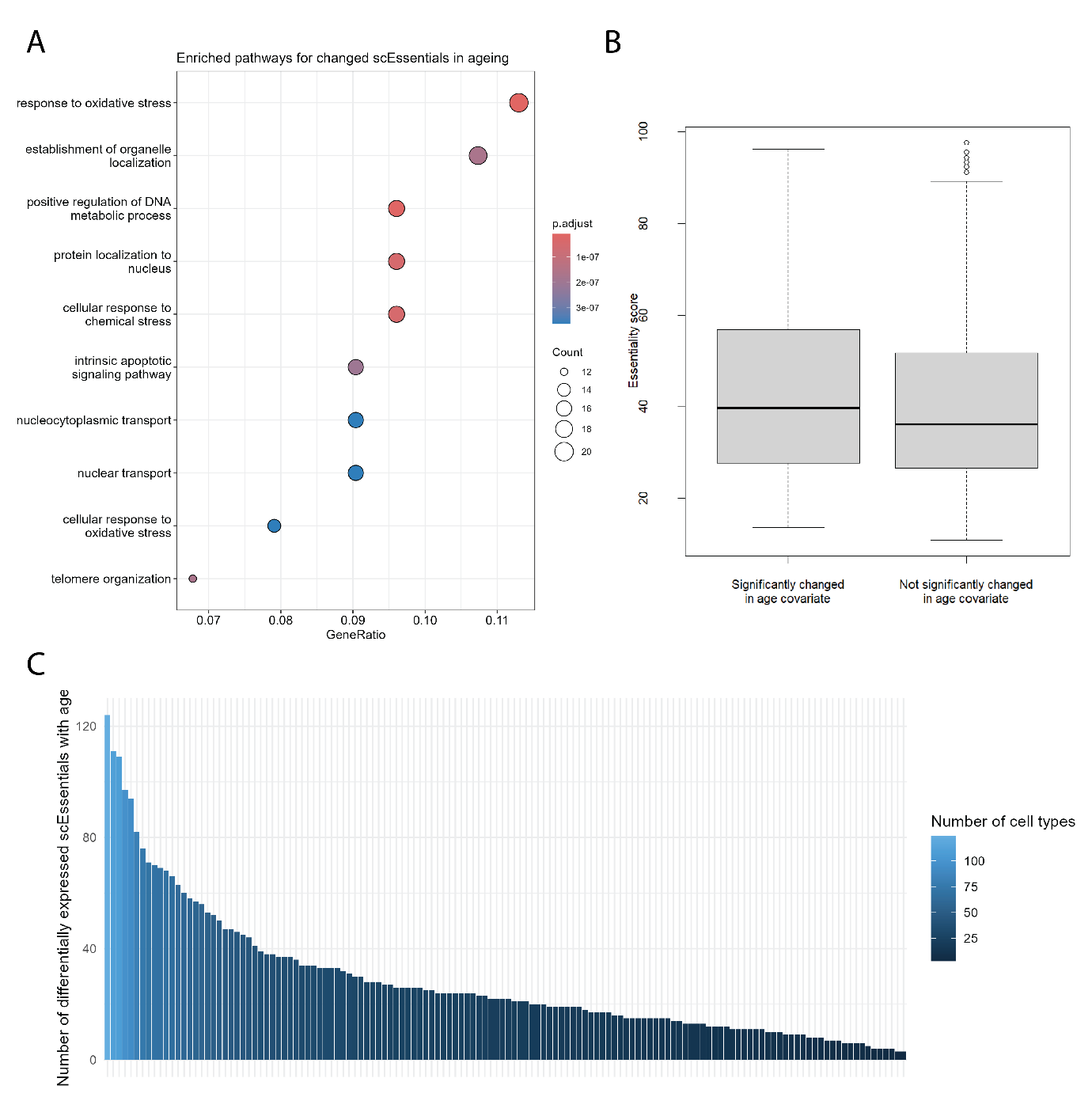


Supplementary Figure 19. Dysregulation of scEssentials genes among different cell types in ageing. A) Dotplot for the top 10 significantly enriched pathways for the scEssentials that changed during age covariates. The analysis was performed with GO database biological processes. B) Boxplot illustrated the significant difference in the essentiality score between significant age covariates and the non-significant age covariate group. Wilcoxon ranked test was applied to determine the significance. C) Barplot showed the number of significantly changed scEssentials across cell types in ageing.

1. Ziegenhain C, Vieth B, Parekh S, Reinius B, Guillaumet-Adkins A, Smets M, et al. Comparative Analysis of Single-Cell RNA Sequencing Methods. Molecular Cell. 2017;65(4):631-43.e4.

2. Chen W, Zhao Y, Chen X, Yang Z, Xu X, Bi Y, et al. A multicenter study benchmarking single-cell RNA sequencing technologies using reference samples. Nature Biotechnology. 2021;39(9):1103-14.

3. Mereu E, Lafzi A, Moutinho C, Ziegenhain C, McCarthy DJ, Alvarez-Varela A, et al. Benchmarking single-cell RNA-sequencing protocols for cell atlas projects. Nat Biotechnol. 2020;38(6):747-55.

4. Jones Robert C, Karkanias J, Krasnow Mark A, Pisco Angela O, Quake Stephen R, Salzman J, et al. The Tabula Sapiens: A multiple-organ, single-cell transcriptomic atlas of humans. Science.376(6594):eabl4896.

5. Hounkpe BW, Chenou F, de Lima F, De Paula Erich V. HRT Atlas v1.0 database: redefining human and mouse housekeeping genes and candidate reference transcripts by mining massive RNA-seq datasets. Nucleic Acids Research. 2021;49(D1):D947-D55.

6. Deeke JM, Gagnon-Bartsch JA. Stably expressed genes in single-cell RNA sequencing. Journal of Bioinformatics and Computational Biology. 2020;18(01):2040004.

7. Lin Y, Ghazanfar S, Strbenac D, Wang A, Patrick E, Lin DM, et al. Evaluating stably expressed genes in single cells. GigaScience. 2019;8(9):giz106.

8. Itan Y, Shang L, Boisson B, Patin E, Bolze A, Moncada-Vélez M, et al. The human gene damage index as a gene-level approach to prioritizing exome variants. Proceedings of the National Academy of Sciences. 2015;112(44):13615-20.
