# Supplementary material for: Assessing tissue-specific gene expression of essential genes from human and mouse
Source: Brief Bioinform. 2025 Sep 24;26(5):bbaf487. doi: 10.1093/bib/bbaf487 (PMC12459261; doi:10.1093/bib/bbaf487)
Supplement: SupplementaryNote_Revised_Clean_26Aug2025_bbaf487 [file supplementarynote_revised_clean_26aug2025_bbaf487.docx]

**Supplementary Note**

**Extended Discussion of the Methods**

*Benchmarking datasets for refining essential genes for scEssentials.*

Single-cell RNA-seq datasets were sourced from mouse and human to evaluate the specificity and reliability of essential gene expression. For specificity, we used the Tabula Muris (TM) and Tabula Sapiens (TS) which are single-cell atlases that cover a broad range of tissue and cell types from mouse and human, respectively. Our study explicitly used the data generated by FACs SmartSeq2 technology since these datasets are less sparse than the Droplet-based options in TM. We used data from the young groups (3 months for TM and < 40 years for TS) with 7 mice and 5 donors, respectively. Only cell types that have more than 100 cells were included, resulting in 68 unique tissue-cell types for TM and 53 unique tissue-cell-types for TS.

Two benchmarking datasets were used to evaluate the reliability of expression by assessing technical variation. The mouse embryonic stem cells (mESCs) that have been sequenced by CELseq2, Dropseq, MARSeq, SCRBseq, Smartseq and Smartseq2 (1) and a matched control ‘normal’ B lymphocyte line from breast cancer patients that have been sequenced by 10x Genomics Chromium, Fluidigm C1, Fluidigm C1 HT and Takara Bio ICELL8 (2) as listed in Supplementary Table 1 (Supplementary Figure 1A & 2A) .

To expand our evaluation process to incorporate more sequencing protocols, we used an additional benchmarking dataset sequenced by a combination of 13 distinct single-cell and single-nuclei sequencing protocols to further ensure the generalisability of scEssentials, including CELseq2, MARSeq, Quartz-seq2, SCRB-seq, Smartseq2, ddSEQ, ICELL8, C1HT-small, C1HT-medium, Chromium, Chromium (sn), Dropseq, and inDrop (3). This dataset features a mixed-species design composed of 60% human PBMCs, 30% mouse colon cells, and 10% cell lines (from human, mouse, and dog) and we focused on the human reference genome mapping and mouse reference genome mapping data, respectively (Supplementary Figure 1C-D & 2C-D).

*Developing a computational method, scEssentials, to assess essential gene expression*

Because single-cell RNA-sequencing experiments typically have higher dropout rates and technical limitations that affect scale, some of these essential genes were not detected in scRNA-seq data. Therefore, we imposed a series of selection criteria to ensure the scEssentials generalisability.

First, we used two benchmarking datasets to ensure the detectability of scEssentials genes across major sequencing platforms, so that for any given essential gene *x_m_* had at least 3 non-zero reads in every sequencing method that was sequenced only with cell line samples and we selected genes from the intersection (Figure 1), denoted as:

$$\prod_{methods} (x_{m}>3)>0$$

Additionally, we also hypothesised that essential genes with excessive expression variations across different sequencing met were more likely to be affected by sequencing technology so that should be removed. To determine highly variable essential genes, we ranked essential genes based on their expression in each sequencing method, where genes with the highe SD had the higher rank. To systematically determine how to remove the genes that were considered unstable essential genes, we tested a range of cutoffs from 1 to 10 times the standard deviation of the ranks (Supplementary Figure 1B & 2B). Between the window of 1 to 10, we identified an elbow point at 4 times the standard deviation and hence this was adopted as the threshold in our approach. As a result, any essential genes with average ranks across sequencing methods that were higher than four times standard deviation of the distribution of the ranks were removed from further analysis, resulting in 1237 and 5770 essential genes for mouse and human, respectively.

*Constructing an Essentiality score*

Given a set of scEssentials genes, the raw essentiality score (ES) for gene $i$ across $k$ cell types is computed as:

$Raw {ES}_{i}=\left( 1-\omega_{i} \right)* \frac{\sum_{k} {pct}_{ik}}{N_{k}}$

where ${pct}_{ik}$ is the percentage of cells that express gene $i$ in cell type $k$. Measuring the ${pct}$instead of mean expression for each gene provided a wider comparison range while maintaining the characteristics of the high average expression given the high correlation between ${pct}_{ik}$ and mean expression (Supplementary Figure 4). $\omega_{i}$ is the cell-type-specificity weight calculated from *PanglaoDB* (version 27_Mar_2020) as well as the frequency of gene $i$ has been identified as a marker in TM and TS, namely,

$$\omega_{i}= \left\{ \begin{aligned} 0 ; S_{i}=0 &\cup G_{i}=0 \\ f\left( i \right); f\left( i \right)= \frac{S_{i}+G_{i}}{2}, G_{i}= \frac{\sum_{k} ({DEG}_{k}\cap i)}{N_{k}} \end{aligned} \right.$$

where $S_{i}$ referred to sensitivity from *PanglaoDB* and$G_{i}$ referred to the number of cell types where gene $i$has been identified as a cell-type-specific DEG marker from the TM or TS dataset. Thus, genes that are less frequently identified as DEGs across different sources and are expressed in a higher number of cells will be upweighted.

Although *PanglaoDB* integrates 1368 single-cell datasets and employs a community-based curation approach to identify gene expression markers across 155 cell types, it has certain limitations. These include biological biases such as uneven representation of tissue types and technical variability stemming from the use of different sequencing platforms. However, given the muti-step filtering criteria applied to define scEssentials, including cross-sample stability and cross-platform robustness, we expect that many of the biases from marker annotation are mitigated.

Furthermore, we show that scEssentials genes have a low overlap with DEGs, suggesting that the sensitivity score, used as a weight in the essentiality score, has limited influence on the final ranking of these genes. To further assess the robustness of our approach, we compared the scEssentials genes using an independent and comprehensive marker resource, *CellMarker 2.0* (4). The results demonstrated consistently low gene overlaps between scEssentials genes and markers from the *CellMarker 2.0* database, indicating that our method is not affected by potential biases in *PanglaoDB* (Supplementary Figure 3).

Notably, the distribution of raw ES was right-skewed (Supplementary Figure 5), with a median ES score at 20. To mitigate the skewness and achieve compatible values, logarithm_2_ transformation was applied to raw ES.

${ES}_{i}={log}_{2} (Raw {ES}_{i})$

*Assessing the significance of the essentiality score relative to a random gene set.*

To characterise the scEssentials, we used random sampling gene lists as the control to compare against. All the random gene list was sampled under the full data matrix with the same number of essential genes and the random sampling was computed 10 times.

*Comparison of scEssentials genes and other reference gene sets*

To further examine the distinct characteristics of scEssentials in relation to SEGs, ribosomal genes, housekeeping genes, and cell type-specific markers, we conducted hypergeometric tests for each pairwise overlap. The total number of genes detected in the TM and TS datasets was used as the background universe for mouse and human comparisons, respectively. False discovery rate (FDR) correction was applied to adjust the resulting p-values.

References

1. Ziegenhain C, Vieth B, Parekh S, Reinius B, Guillaumet-Adkins A, Smets M, et al. Comparative Analysis of Single-Cell RNA Sequencing Methods. Molecular Cell. 2017;65(4):631-43.e4.

2. Chen W, Zhao Y, Chen X, Yang Z, Xu X, Bi Y, et al. A multicenter study benchmarking single-cell RNA sequencing technologies using reference samples. Nature Biotechnology. 2021;39(9):1103-14.

3. Mereu E, Lafzi A, Moutinho C, Ziegenhain C, McCarthy DJ, Alvarez-Varela A, et al. Benchmarking single-cell RNA-sequencing protocols for cell atlas projects. Nat Biotechnol. 2020;38(6):747-55.

4. Hu CX, Li TY, Xu YQ, Zhang XX, Li F, Bai J, et al. CellMarker 2.0: an updated database of manually curated cell markers in human/mouse and web tools based on scRNA-seq data. Nucleic Acids Research. 2023;51(D1):D870-D6.
